# Extracellular Matrix Components and Mechanosensing Pathways in Health and Disease

**DOI:** 10.3390/biom14091186

**Published:** 2024-09-20

**Authors:** Aikaterini Berdiaki, Monica Neagu, Petros Tzanakakis, Ioanna Spyridaki, Serge Pérez, Dragana Nikitovic

**Affiliations:** 1 Department of Histology-Embryology, Medical School, University of Crete, 712 03 Heraklion, Greece; berdiaki@uoc.gr (A.B.); petzanakak@gmail.com (P.T.); ispyridaki@uoc.gr (I.S.); 2 Immunology Department, “Victor Babes” National Institute of Pathology, 050096 Bucharest, Romania; monica.neagu@ivb.ro; 3 Centre de Recherche sur les Macromolécules Végétales (CERMAV), Centre National de la Recherche Scientifique (CNRS), University Grenoble Alpes, 38000 Grenoble, France; serge.perez@cermav.cnrs.fr

**Keywords:** mechanotransduction, proteoglycans, glycosaminoglycans, mechanosensing, cancer, inflammation, syndecans, glypican

## Abstract

Glycosaminoglycans (GAGs) and proteoglycans (PGs) are essential components of the extracellular matrix (ECM) with pivotal roles in cellular mechanosensing pathways. GAGs, such as heparan sulfate (HS) and chondroitin sulfate (CS), interact with various cell surface receptors, including integrins and receptor tyrosine kinases, to modulate cellular responses to mechanical stimuli. PGs, comprising a core protein with covalently attached GAG chains, serve as dynamic regulators of tissue mechanics and cell behavior, thereby playing a crucial role in maintaining tissue homeostasis. Dysregulation of GAG/PG-mediated mechanosensing pathways is implicated in numerous pathological conditions, including cancer and inflammation. Understanding the intricate mechanisms by which GAGs and PGs modulate cellular responses to mechanical forces holds promise for developing novel therapeutic strategies targeting mechanotransduction pathways in disease. This comprehensive overview underscores the importance of GAGs and PGs as key mediators of mechanosensing in maintaining tissue homeostasis and their potential as therapeutic targets for mitigating mechano-driven pathologies, focusing on cancer and inflammation.

## 1. Introduction

The survival of all living organisms depends on their ability to adapt to environmental stresses, particularly mechanical forces. These forces are essential for the formation, development, and maintenance of tissues and organs. Organisms have evolved structures at various levels, including organs, tissues, cells, and molecular assemblies, that can sense and respond to various forces, such as compression, tension, shear, and hydrostatic pressure.

Cells, the fundamental blocks of life, possess intricate mechanisms that enable them to sense and respond to mechanical signals in their environment. Their remarkable adaptability and resilience are demonstrated in how cells interpret and respond to these cues, a process known as mechanosensing. In addition, cells can adapt to these signals through changes in signaling pathways and gene expression, a phenomenon known as mechanotransduction [1]. These mechanical signals can originate from the rigidity of the surrounding substrate and neighboring cells and fluid flow on cells lining blood vessels [2,3]. The complexity of these cellular mechanisms is a testament to the intricacy of life itself.

Cellular responses to force can be highly variable, including changes in cell positioning, morphology, dimensions, proliferation, adhesion, and stiffness. They can also include changes in intracellular trafficking, secretion patterns, gene expression, and chromatin structure [4]. Therefore, understanding how cells sense, interpret, respond to, and adapt to their mechanical environment is paramount. This involves identifying specific molecular components, understanding their interactions, and elucidating the principles that govern mechanosensing mechanisms and potentially modifying them for particular purposes [5]. This review focuses on the role of PGs and GAGs, critical components of the ECM, in mechanotransduction.

## 2. The Interplay between the ECM and Its Specific Component, the Cellular Glycocalyx (GCX)

**Figure 1:**
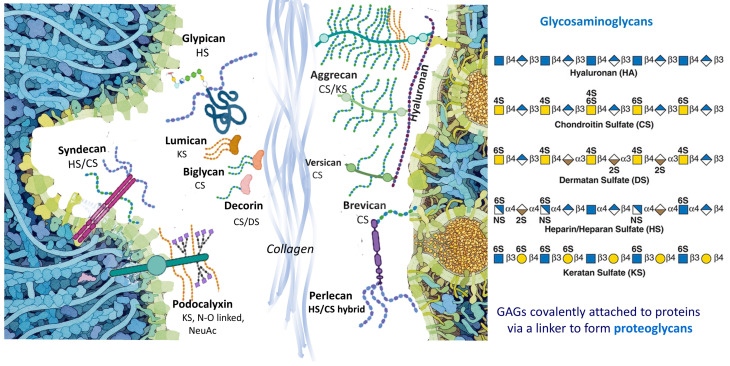
Schematic representation of the major proteoglycan constituents of the extracellular matrix (ECM) and the cellular glycocalyx (GCX) (adapted from a depiction presented in [7]). The structures of the glycosaminoglycan components are displayed. **HA**, hyaluronan: 4-D-GlcA-β1-3-D-GlcNAc-β1. **CS**, (4S/6S)-chondroitin 4/6-sulfate: 4-D-GlcA-β1-3-D-GalNAc, 4S/6S-β1. **DS**, Dermatan sulfate: -4-L-IdoA-α1-3-DGalNAc, 4S-β1-. **HS**, heparan sulfate: -4-D-GlcNAc-α1, 4-D-GlcA-β1-. **KS**, keratan sulfate: -4-D-GlcNAc, 6S-β1-3-D-Gal-β1-. Color displayed in the monosaccharide units follows the SNFG recommendations. Perez, Serge; Nikitovic, Dragana (2024). Proteoglycans extracellular matrix. figshare. Figure: https://doi.org/10.6084/m9.figshare.26963677.v2 (accessed on 9 September 2024). CC by 4.0.

The mechanical properties of the ECM play a fundamental role in determining the structure, function, and behavior of tissues. The ECM is found outside the cells and fills the space between tissue cells. It is prominent in connective tissues such as bone and cartilage. In organs, it is the interstitial matrix. Specifically, the ECM is a complex network of proteins, glycoproteins, and polysaccharides that physically supports cells and tissues and facilitates various cellular processes such as adhesion, migration, and differentiation. It is a scaffold that provides structural integrity and regulates cellular behavior, signaling, and tissue homeostasis. Consisting of a diverse array of proteins, carbohydrates, and other molecules, the composition of the ECM reflects its multifunctional role in various physiological processes, including development, wound healing, and tissue repair. The cellular glycocalyx (GCX), located on the outer surface of the cell membrane, is a unique compartment of the ECM that strengthens the external barrier of a cell and regulates mechanotransduction and growth factor signaling. It is particularly prominent in endothelial cells lining blood vessels and epithelial cells lining body cavities [6]. See Figure 1.

### 2.1. Collagens

Collagens, fibrous proteins with a unique triple-helix structure, are the most abundant proteins in the ECM and fundamental to its architecture. Each collagen molecule consists of three polypeptide chains, called alpha chains, intertwined like a rope. These alpha chains are rich in glycine, proline, and hydroxyproline residues, giving the collagen triple helix distinctive stability and properties. This structure provides tissues with tensile strength and elasticity, essential for skin integrity, tendons, ligaments, and bones. Collagens are classified into different types based on their amino acid sequences and distribution in different tissues, highlighting their specialized functions at different stages of development. Types range from collagen type I to XXVIII, highlighting their diverse roles in maintaining tissue health [8].

### 2.2. Elastin

Elastin, together with collagen, is a fibrous protein in the ECM, particularly in tissues that require elasticity and recoil properties. This highly elastic protein is responsible for providing tissues with elasticity and flexibility. Its primary structure consists of repetitive sequences augmented in hydrophobic amino acids such as glycine and alanine, interspersed with cross-linking domains containing lysine residues. The formation of cross-linked networks by elastin molecules allows tissues to stretch and recoil without undergoing permanent deformation. Elastin fibers allow organs, such as the skin, lungs, and blood vessels, to stretch and contract, providing functional resilience and flexibility. The balance between collagen and elastin is instrumental in determining the mechanical properties of tissues, ultimately influencing their ability to withstand deformation and maintain structural integrity [9].

### 2.3. PGs

PGs are a class of glycosylated proteins widely expressed in various tissues and play an essential role in cellular interactions and signaling events (reviewed in [10]). In addition to being a major component of the mammalian GCX, they are also an essential component of the ECM and are also present intracellularly and pericellularly. Their significance in these different contexts underlines their importance in cellular function and homeostasis. The vast majority of PGs are characterized by at least one covalently linked GAG chain, with the exception of some small leucine-rich proteoglycans (SLRPs), which have been detected as non-glycosylated protein cores. PGs may also contain other N- and O-linked glycans found in glycoproteins or glycolipids. Their GAG chains have very distinct glycosylation patterns, consisting of repeating disaccharide units that are long and sulfated at different points of the GAG chain and each monosaccharide. To date, forty-five PGs have been identified, and each member is characterized by immense variability due to the modifications of the protein core and by the type and different stoichiometry of the GAG chain substitutions.

PGs such as syndecans, glypicans, perlecan, betaglycan, and versicans are integral components of the GCX, each contributing to various cellular processes, including adhesion, signaling, and structural integrity. These PGs interact with ECM components, growth factors, and cell surface receptors, highlighting their importance in maintaining cellular functions and responses.

#### 2.3.1. Syndecans and Glypicans

Syndecans and glypicans are prominent families of cell surface PGs. Syndecans are transmembrane PGs with a core protein that spans the cell membrane. They are involved in cell adhesion, cytoskeletal organization, and signal transduction. They bind to the ECM components and growth factors, thereby influencing cell behavior and communication. On the other hand, the glypican family members are connected to the cell membrane via a glycosylphosphatidylinositol (GPI) anchor [10,11,12]. They regulate growth and differentiation by modulating signal pathways. Their HS chains interact with growth factors, enzymes, hedgehog proteins, wingless-related integration site (Wnt) ligands, and other extracellular molecules [10]. Betaglycan, also known as transforming growth factor beta (TGFβ) receptor III, is a core protein with attached GAG chains, primarily CS and HS. It acts as a co-receptor for TGFβ, modulating its availability and activity. It influences cell growth, differentiation, and synthesis of ECM components.

#### 2.3.2. Pericellular PGs

Pericellular PGs, such as perlecans, are predominantly found in the basement membrane. Perlecans, large HSPGs, are found primarily in basement membranes. They are essential for maintaining the integrity of the basement membrane and regulating cell adhesion, proliferation, and differentiation. Perlecan binds to various growth factors and assists the filtration processes, particularly in the kidney glomeruli. Agrin is a PG similar to perlecan, with both HS and CS GAGs.

### 2.4. GAGs

There are five major sulfated GAG chains: heparin (Hep), CS, dermatan sulfate (DS), keratan sulfate (KS), HS, and the non-sulfated hyaluronan (HA). They are linear, long-chained polysaccharides with repeating disaccharide units linked by glycosidic bonds. These units are composed of N-acetylated hexosamine (GlcNAc and GalNAc) and uronic acid (IdoA and GlcA). The type of the disaccharide repeating unit and its modifications, including discrete sulfation patterns, allows GAGs to be classified into specific categories, e.g., Hep/HS, CS/DS, KS and HA. KS chains contain galactose (Gal) instead of uronic acid in their disaccharide building blocks. CS/DS, HS/Hep, and KS chains are covalently linked to the protein cores of PGs. Each GAG is made up of specific monosaccharide building blocks. For example, Hep includes GlcNAc, IdoA, and GlcAc [12], CS is composed of GalNAc and GlcA, and DS comprises IdoA and GalNAc. KS consists of galactose (Gal) and GlcNAc [13]. HS contains GlcNAc, GalNAc, IdoA, and GlcA [14]. The differences in the composition of individual GAGs, expressed on PGs, have been determined by sequencing for most GAGs except for KS. These alterations exert profound effects on GAG function inside and outside the cell. On the other hand, the non-sulfated GAG HA is not bound into the PG core, but is secreted into the ECM of almost all tissues [13].

### 2.5. Glycoproteins

Glycoproteins contain carbohydrate chains that are often branched and complex in structure and are covalently linked to specific amino acid residues within the protein backbone. Glycoproteins, including fibronectin, laminin, and thrombospondin, are essential components of the ECM involved in cell adhesion, migration, and signaling. Fibronectin and laminin have specific protein domains that interact with cell surface receptors, promoting cell adhesion, migration, and signaling. Their carbohydrate moieties fine-tune their affinities to cell surface receptors. Fibronectin, for example, plays a pivotal role in wound healing by facilitating cell migration and adhesion to the ECM, orchestrating tissue remodeling processes [15].

### 2.6. Cross-Linking Molecules

Cross-linking molecules, such as lysyl oxidase and transglutaminases, contribute to ECM stability and organization by forming covalent bonds between ECM components. These cross-links increase the mechanical strength and resilience of tissues, ensuring structural integrity and resistance to degradation. In particular, lysyl oxidase catalyzes the cross-linking of collagen and elastin fibers, oxidizing lysine and hydroxylysine residues, thereby imparting tensile strength and stability to the ECM network. On the other hand, transglutaminases catalyze the formation of cross-links between glutamine and lysine residues in various ECM proteins, further contributing to ECM stability and organization.

### 2.7. The ECM as a Unit

The ECM is essentially a hydrogel in which I and III collagens form the primary structural fibers, with tropocollagen molecules assembling into fibers through covalent and electrostatic interactions, characterized by a 67 nm stagger. This assembly gives rise to the banding pattern of collagen fibers in electron micrographs and Bragg reflections in X-ray scattering [16]. These fibers create a cross-linked network that provides tensile strength to the ECM and serves as a scaffold for GAG and PG molecules. Non-fibrillar type IV collagen, found in basement membranes, aids in anchoring PG molecules in a similar way to type I and III collagens [17]. PG protein cores typically have a molecular weight (MW) of around 20 to 450 kDa [10]. These large molecules are effectively trapped within the collagen network, where the negatively charged carboxyl and sulfate groups create a defined electric charge within the ECM. This constant charge acts like an osmotic sponge, allowing polysaccharide molecules to retain significant amounts of water within the ECM. This property is critical to the elasticity of the ECM, as the presence of incompressible water, osmotically held by the polysaccharides, limits water outflow under mechanical compression, thereby enabling the tissue to resist compression [18]. Moreover, this mechanism allows tissues such as cartilage to distribute loads and recover shape after deformation. In cartilage, for example, the regulation of water flow is essential for shock absorption during joint movement, where the interplay between ECM components and water determines the tissue’s ability to handle repeated mechanical stresses. Alterations in water content, often seen in pathological conditions like osteoarthritis, can lead to compromised tissue function and reduced mechanical resilience [18].

## 3. Several Critical Mechanical Properties of the ECM Contribute to Its Functionality

### 3.1. Stiffness/Elasticity

The stiffness or elasticity of the ECM, often referred to as its “mechanical stiffness”, is a critical determinant of cell behavior. Cells can sense and respond to the mechanical properties of their environment through mechanotransduction mechanisms. Changes in ECM stiffness can influence cellular processes, such as proliferation, differentiation, and gene expression. The stiffness and elasticity of the ECM are primarily determined by the composition and organization of its molecular components. Several vital molecules, such as collagen, elastin, PGs, glycoproteins, matrix metalloproteinases (MMPs), cross-linking molecules, and water, confer crucial mechanical properties to the ECM [19]. Overall, the intricate interplay between these molecular components determines the mechanical properties of the ECM, including its stiffness, elasticity, and viscoelastic behavior. Dysregulation of ECM molecules can lead to alterations in tissue mechanical properties, contributing to various pathological conditions such as fibrosis, arthritis, and cardiovascular disease.

### 3.2. Viscoelasticity

The ECM has both elastic (recoverable deformation) and viscous (non-recoverable deformation) properties, known as viscoelasticity. This property allows the ECM to deform under applied forces and return to its original state when the force is removed. Viscoelasticity is essential for tissues subjected to dynamic mechanical forces, such as blood vessels and tendons. The viscoelastic properties of the ECM are influenced by several molecular components that contribute to its dynamic behavior in response to mechanical forces. Some of the critical molecules involved in conferring viscoelasticity to the ECM are PGs, such as aggrecan and decorin, as well as GAGs, including HA, CS, and HS. These molecules interact with collagen and elastin fibers to give the ECM its viscoelastic properties [20,21].

### 3.3. Strength and Toughness

The ECM offers structural support and mechanical strength to tissues, enabling them to withstand mechanical stress and strain. The strength of the ECM is determined by the composition and organization of its components, including collagen fibers and PGs. Toughness refers to the ability of the ECM to absorb energy and resist fracture or deformation under stress. Several critical molecules that impart strength and toughness to the ECM are collagen, fibronectin, elastin, versican, elastin, and laminin

### 3.4. Anisotropy

The ECM often exhibits anisotropic mechanical properties, meaning its mechanical behavior varies with direction. This property is fundamental in tissues with specialized functions and complex hierarchical structures, such as bone and cartilage. Anisotropy allows tissues to withstand mechanical loads along specific orientations. The anisotropic mechanical properties of the ECM, which refer to variations in the mechanical behavior depending on the direction of applied forces, are influenced by several molecular components. These molecules contribute to the specialized organization and structural alignment within the ECM, resulting in directional differences in mechanical properties. Critical molecules conferring anisotropic mechanical properties to the ECM are HA and CS/DS [22].

### 3.5. Adhesive Properties

The ECM provides a substrate for cell adhesion and migration through adhesive proteins such as fibronectin, laminin, and integrins. The adhesive properties of the ECM influence cell spreading, morphology, and signaling, thereby regulating various cellular functions. The adhesive properties of the extracellular matrix are crucial for cell attachment, migration, and signaling. Several molecules within and associated with the ECM are essential in mediating cell–ECM interactions. Fibronectin, laminin, integrins, GAGs, PGs, tenascin, and collagen are key molecules involved in the adhesive and mechanical properties of the ECM [23,24,25].

### 3.6. Remodeling and Plasticity

The ECM is dynamic and undergoes continuous remodeling in response to mechanical and biochemical stimuli. This remodeling process, mediated by cells such as fibroblasts and MMPs, allows tissues to adapt to changing mechanical environments and repair damage. The ability of the ECM to remodel is regulated by several molecular components (Table 1), which contribute to tissue homeostasis, repair, and adaptation to physiological and pathological conditions. Dysregulation of ECM remodeling processes has been implicated in various diseases, highlighting the importance of understanding the molecular mechanisms underlying ECM plasticity.

In addition, blood, a highly specialized form of fluid tissue, exerts shear stress on the endothelial cells that line blood vessels. The endothelial cells’ apical surface is covered by a GCX, which plays a crucial role in sensing external signals and controlling vascular permeability and barrier functions. Researchers often study the apical and basal GCX separately because they are exposed to different stimuli. The apical GCX senses fluid shear forces and transmits them into the cell by linking to the cytoskeleton, while the basal GCX responds to shear stress induced by blood flow, leading to distinct signaling pathways [26]. The mechanistic aspects of shear stress transduction and the involvement of PG/GAG are also discussed.

## 4. Receptors Involved in the Process of Mechanotransduction

### 4.1. Integrins

Integrins are heterodimeric transmembrane receptors that link the ECM to the cytoskeleton and mediate cell–cell and cell–ECM interactions. Active integrin receptors consist of obligate heterodimers, comprising an α-subunit and a β-subunit. Although these molecules are substantially dissimilar in sequence, they share the common feature of being type I transmembrane proteins. They have large extracellular domains, a single-pass transmembrane segment, and typically short cytoplasmic tails. Mammals have 18 α-subunits and eight β-subunits, which can combine to form 24 different αβ heterodimers. They play critical roles in mechanotransduction by transmitting mechanical forces across the cell membrane and modulating intracellular signaling pathways (mechanosensing) [27,28].

Indeed, at adhesion sites, integrins link the ECM to the F-actin cytoskeleton, transmitting mechanical forces from actin/myosin II coupling to the ECM through mechanosensitive focal adhesion proteins. This force transmission creates mechanical reciprocity between the ECM’s viscoelasticity and cellular tension. During mechanotransduction, forces modify mechanosensitive proteins within adhesions, triggering biochemical signals that control immediate cellular mechanics and long-term gene expression changes [27,28].

Specifically, upon activation, integrins couple dynamically to the actomyosin system through integrin- and/or F-actin-binding proteins, including vinculin and talin [29]. This mechanical connection is typically denoted the molecular clutch [30]. Talin directly connects the cytoplasmic domain of activated integrins to F-actin. Talin boasts an N-terminal FERM domain, also known as the talin head domain (THD), consisting of four-point-one, ezrin-, radixin-, and moesin-binding sites, a long C-terminal rod domain with 13 helical bundles (R1–R13), and a dimerization motif. The THD interacts with the membrane-proximal NPxY motif of β-integrin tails, negatively charged lipids in the plasma membrane, and the cortical F-actin network (actin-binding site 1 [ABS1]). The rod domain contains two more F-actin-binding sites (ABS2 and ABS3), 11 vinculin-binding sites (VBSs), and binding sites for regulatory proteins like RIAM and Kank family proteins, as well as Rho GTPase-activating protein DLC1 [30,31,32]. The integrin-F–actin linkage is further reinforced by vinculin, which binds to both talin and F-actin.

Additionally, proteins like kindlin and α-actinin may also play a role in the molecular clutch created by the dynamic associations between integrins bound to the ECM and the force-generating actomyosin cytoskeleton. Kindlins likewise contain an FERM domain, which binds to a discrete form of talin, and a β-integrin cytoplasmic domain to modulate integrin activation [33]. However, in contrast to talins, kindlins are not able to modulate the conformation of the integrin transmembrane helix and activate integrins independently [34]. Alpha-actinin is a cytoskeletal actin-binding protein that forms an anti-parallel rod-shaped dimer with actin-binding domains at both ends. It contributes to the organization of actin filaments in various cell types and cytoskeletal frameworks. In non-muscle cells, alpha-actinin is present along actin filaments and at adhesion sites.

This mechanical connection is a highly adaptable system that responds sensitively to the multiple levels of ECM rigidity. Thus, on stiffer substrates, rapid mechanical loading rates on talin cause partial protein unfolding and reveal hidden vinculin-binding sites (VBS). Vinculin binding to talin then strengthens the molecular clutch, enhancing force transmission. On soft ECM substrates, which deform easily, the slow mechanical loading rates on talin are insufficient to trigger vinculin-dependent reinforcement, maintaining a low-level transition of force [30,31,32].

### 4.2. Cadherins

Cadherins are calcium-dependent cell adhesion molecules capable of transmitting mechanical forces between cells and thus mediating cell–cell adhesion and tissue morphogenesis. They participate in mechanotransduction and regulation of cell signaling pathways [35]. Their structure features an extracellular domain with five beta-barrel regions, each with three calcium-binding sites at the junctions. These extracellular domains are numbered one through five, starting with the N-terminal domain, which includes the main adhesion site. Recent studies employing various experimental methods have shown that cadherin adhesion involves multiple distinct cadherin–cadherin bonds. These bonds engage different structural regions and display varying kinetic and mechanical properties [36]. Cadherins also boast a single-pass transmembrane domain and a cytoplasmic domain that interacts with various proteins involved in signaling and the formation of the cytoskeleton cytoplasmic signaling and cytoskeletal proteins [37].

Catenins, including α-, β-, γ-, and p120 catenin (p120ctn), are key cadherin cytoplasmic binding partners linking the receptors to the actomyosin network. Both β-catenin and γ-catenin bind to cadherin cytodomains, connecting the complex to the actin cytoskeleton and potentially to intermediate filaments via linker proteins [37]. Cadherin bonds convey mechanical information to cells by resisting forces from endogenous contraction or external sources like fluid shear, tissue rigidity, and compression. While cadherin ligation alone can trigger biochemical signaling, cadherin complexes also link the cytoskeletons of neighboring cells, forming a mechanical chain that responds to changes in tensile forces due to dynamic cytoskeletal deformations [38]. Thus, cadherin-dependent mechanotransduction involves force-dependent remodeling of intercellular junctions, changes in mechanical properties such as cell traction and adhesion, and alterations in junction stiffness.

The pivotal molecule in cadherin-dependent mechanotransduction is α-catenin, which connects E-cadherin-associated β-catenin to F-actin [39]. Upon α-catenin binding, a vinculin-binding site on E-cadherin is revealed, allowing vinculin mobilization [40]. In addition to vinculin, α-actinin also binds to cadherin, regulating actin-related protein 2/3 complex (ARP2/3) activity to assemble and sustain F-actin at E-cadherin junctions connecting cadherin mechanotransduction to F-actin polymerization [41].

### 4.3. PIEZO and TRP

PIEZO and TRP are mechanosensitive ion channels that open or close in response to mechanical stimuli, allowing ions to flow across the cell membrane. They mediate several physiological processes, including touch sensation, proprioception, and vascular regulation [42]. PIEZO1 is critical for the cellular perception of mechanical forces and triggers inflammation in response to stress. The triskelion structure of PIEZO channels allows them to be directly activated by membrane stretch, with PIEZO1 reaching half-maximal activation at pressures of −27  ±  3.4 mmHg. In addition, interactions with cellular components such as the ECM proteins, actin cytoskeleton, cell membrane lipid composition, and integrins can modify the activation threshold. Among others, PIEZO1 converts mechanical stimuli into pro-inflammatory signals and plays a central role in chronic inflammatory diseases such as Alzheimer’s disease, myocardial fibrosis, atherosclerosis, osteoarthritis, and lumbar degeneration [43]. Members of the TRP channel family, such as TRPV4, TRPC6, and TRPM7, are implicated in mechanotransduction and are involved in sensing mechanical stimuli in various cell types and tissues [44].

### 4.4. G Protein-Coupled Receptors (GPCRs)

GPCRs: Some GPCRs are sensitive to mechanical forces. When activated by mechanical stimuli, they can initiate intracellular signaling cascades. Examples include the endothelial cell GPCR GPR68 or OGR1 (ovarian cancer G protein-coupled receptor 1). GPR68 is a GPCR that responds to extracellular acidic pH and mechanical stimuli. It is implicated in various physiological processes, including regulating vascular tone and cell proliferation [45].

**Figure 2:**
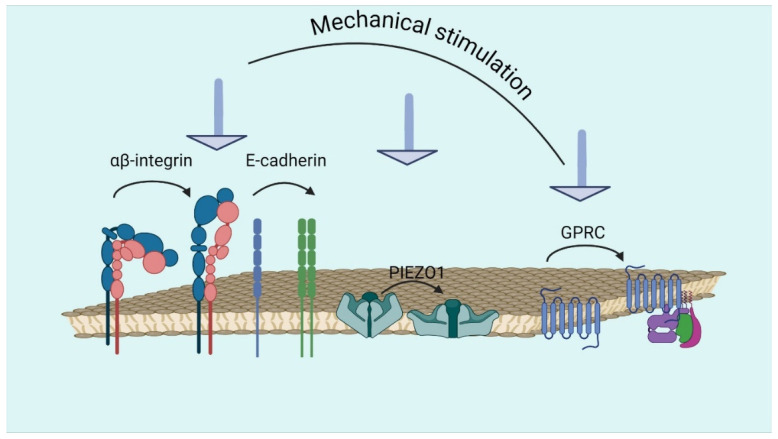
Integrins, cadherins, PIEZO, and GPRC receptors are involved in transmitting mechanical cues from the extracellular space to cells. Mechanical stimulation activates the receptors. Created in BioRender. Nikitovic, D. (2024) BioRender.com/g48k171 (accessed on 12 September 2024).

Recently, a novel mechanism of mechanical GPCR activation in endothelial histamine H1 receptors (H1Rs) triggered by shear stress and membrane stretch was identified. This activation was found to be agonist-independent, unaffected by inverse agonists, and dependent on G11/q-proteins, leading to NO production. Unlike agonist-induced changes, shear stress and stretch caused unique conformational shifts. Notably, this mechanically activated pathway required the eighth helix in the receptor’s C-terminal tail. These findings suggest that the mechanical elongation of H8 activates the G protein [46]. The most common mechanoreceptors are depicted in Figure 2.

## 5. Mechanisms of Mechanosensing

### 5.1. Overview of Mechanosensitive Pathways in Cells

Mechanotransduction pathways are complex, involving multiple molecules and mechanisms. Cells adhere to the ECM via the extracellular domains of the transmembrane integrin receptors, forming nascent adhesions and focal complexes. Nascent adhesions are short-lived adhesions formed during early cell spreading. They contain only a few integrin dimers bound to their ECM ligands [47]. Some nascent adhesions stabilize and recruit more integrins, which bind through their cytoplasmic tails to cytosolic proteins such as talin, transferring focal adhesion complexes. Within these complexes, proteins, including talin, zyxin, vinculin, and alpha-actinin, bind to the F-actin network, transferring forces from the cytoplasm through integrins to the ECM, resulting in traction stress. This stress enables cells to adhere to and move along the ECM [28]. Tensile forces are required for the maturation of nascent adhesions into focal adhesions. However, it is also influenced by the physical properties of the ECM, as cells only form small, short-lived focal adhesions on soft substrates [48]. On a rigid substratum, tension mediated by the activity of the small GTPase RhoA promotes focal adhesion growth and recruitment of multiple proteins to these structures, which can generate more tension in a positive feedback loop [49]. Indeed, RhoA acts as a molecular switch, alternating between an inactive GDP-bound “off” state and an active GTP-bound “on” state. When bound to GTP, RhoA becomes active and interacts with downstream effectors. It then hydrolyzes GTP to GDP, reverting to its inactive state [50].

When subjected to mechanical changes such as stretching of the ECM, cells transmit force to the cytoskeleton via integrin receptors, talin, and other linker proteins to the cytoskeleton. The forces generated can unfold talin and other adhesion proteins, exposing binding sites for F-actin and vinculin. This leads to an increased concentration of proteins at adhesion sites, including enzymes such as focal adhesion kinase (FAK), and further binding to the cytoskeleton [51]. FAK is a multidomain protein with a central kinase domain flanked by an N-terminal FERM domain and a C-terminal FAT domain. In its inactive state, FERM and kinase domains interact, keeping the active site sequestered. Upon integrin-mediated adhesion, the FAT domain directs FAK to focal adhesions, where it associates with the plasma membrane. PIP2 binding to the FERM domain exposes the autophosphorylation site. Autophosphorylation then recruits Src kinase, which activates FAK by phosphorylating its activation loop [52]. The clustering of integrins eventually leads to mature focal adhesion sites consisting of adhesion receptors, signaling molecules, and cytoskeletal proteins [53]. Indeed, focal adhesion complexes are intricate multi-protein assemblies with varied overall structures and protein compositions tailored to their specific functions and environments and include over two hundred distinct proteins [54]. FAK activation controls the turnover of the focal adhesion complexes and cell motility, but also perpetrates cross-talk to modulate cell survival and growth [55].

Firstly, this reinforces adhesion, a typical cell response to external mechanical force, which can trigger cell-wide adaptations. These adaptations can increase traction on the ECM, leading to ECM remodeling through force-induced unfolding of ECM proteins. In addition, force applied to integrin receptors activates the Rho signaling pathway, further enhancing actomyosin force generation. Integrin forces activate enzymes such as Src kinase and modulate signaling pathways through ion channels. The ECM–integrin–cytoskeleton junction is critical for sensing and translating changes in ECM stiffness into intracellular responses [54].

ECM stiffness profoundly influences cell behavior and tissue structure, with a soft ECM resulting in reduced adhesions and traction forces, leading to decreased cell spreading and altered nuclear morphology. The mechanotransduction pathway involving yes-associated protein (YAP)/transcriptional coactivator [56] with PDZ-binding motif (TAZ) translocation to the nucleus is particularly sensitive to mechanical stimuli, independently of the Hippo pathway, highlighting its importance in the cellular response to mechanical cues [57].

The compartmentalization of the ECM adds an additional layer of complexity to mechanosensing. For example, in many tissue types, the immediate cellular microenvironment, known as the “pericellular matrix” or, in the case of lumen-lining cells, the “GCX layer”, has a different composition and structure from the bulk ECM. Such tissues include articular cartilage, meniscus, intervertebral disk, injured tendon, endothelium, stem cell niche, and solid tumors [58]. This specialized microenvironment is characterized by the exclusive localization or preferential distribution of PG and PG-HA complexes. The pericellular matrix PGs are involved in a wide range of interactions with cell surface receptors, growth factors, and cytokines, playing central roles in mediating cellular metabolism, signaling, and cell–matrix communication [59]. In addition, they impart cells with a highly negatively charged environment, which serves as a critical biophysical cue governing cell mechanosensing in vivo [60].

### 5.2. Role of GAGs and PGs in Mechanotransduction

PGs and GAGs can affect cell mechanotransduction through several pathways: (i) acting as mechanoreceptors directly affecting the affinities of integrin and cadherin receptors to ECM and cell membrane ligands; (ii) modulating the physicochemical properties of the ECM; and (iii) affecting the response to shear stress exerted by blood fluid on the endothelial cells.

#### 5.2.1. PGs and Cells

Syndecans, a subset of four HSPGs, feature three main domains: an N-terminal polypeptide to which GAG chains attach, a single transmembrane domain, and a C-terminal cytoplasmic domain [61]. The ectodomains of syndecans facilitate various cell–cell and cell–matrix interactions via their GAG chains. Notably, while the extracellular regions of syndecans vary considerably within family members, the transmembrane and cytoplasmic domains remain highly conserved. Mammals possess four distinct syndecan members. Syndecans interact with components of the ECM, such as fibronectin, collagen, and laminin, and with cell surface receptors, such as integrins. These interactions form a complex network known as the pericellular matrix, which facilitates the transmission of mechanical signals from the ECM to the interior of the cell [62].

Indeed, syndecan 4 appears to exhibit a role similar to integrins in binding to ECM components [63]. Syndecans have been characterized as obligatory non-canonical components of the focal adhesion complexes [53]. Thus, integrins and syndecan 4 have binding sites to the ECM protein vinculin. Single-molecule force spectroscopy showed that the detachment of αVβ1 from vinculin occurs before the detachment of syndecan 4 [64]. Despite their distinct detachment rates, access to both receptors is essential for cell growth. The same methodology demonstrated that syndecan 4 and α5β1 integrin showed resilience in their interactions with fibronectin, even under deformation [65]. In a separate study, tension applied to syndecan 4 led to widespread activation of the kindlin 2–β1 integrin–RhoA axis in a phosphatidylinositol 3-kinase (PI3K)-dependent manner with the participation of the endothelial growth factor receptor (EGFR). In addition, tension mediated by syndecan 4 at the cell–ECM interface is crucial for activating yes-associated protein. Syndecan 4 undergoes a conformational change in its cytoplasmic domain in response to extracellular tension, with the variable region playing a critical role in adapting to mechanical force. This facilitates the formation of a syndecan 4–α-actinin–F-actin molecular scaffold at the adhesion site [66].

In addition, syndecan 4 increases the lifespan of Thy-1–αVβ3 integrin by binding directly to Thy-1 and forming a ternary complex (Thy-1–αVβ3 integrin + syndecan 4) that retracts neurite outgrowth upon force application [67]. Syndecan 4 has also been shown to compensate for the lack of integrin α11 in the heart of α11-knockout mice [68].

In particular, the phosphorylation of syndecan 4 serves as a key regulator of integrin recycling. Src kinase phosphorylates syndecan 4, facilitating its interaction with syntenin, which in turn suppresses ADP-ribosylation factor 6 (Arf6) activity and promotes the recycling of αVβ3 integrin to the plasma membrane while reducing the presence of α5β1 integrin. This shift towards αVβ3 engagement increases the stability of focal adhesions. Conversely, inhibition of syndecan 4 phosphorylation increases surface expression of α5β1 integrin, leading to destabilization of adhesion complexes and impaired cell migration [69].

The use of engineered biomaterials showed that the functionalization with both integrin and syndecan 4 ligands resulted in more significant and faster cell capture under laminar shear flow conditions than surfaces coated with integrin- or syndecan-binding ligands alone. However, both types of ligands were essential for orienting the cells in the direction of the flow. These findings suggest that while integrin engagement is critical for adhesion strength, the involvement of both receptor types is beneficial for effective mechanotransduction [70]. Similarly, artificial matrices have shown that fibroblasts rapidly enhance the initiation and reinforcement of adhesion when exposed to fibrillar fibronectin matrices through α5β1 integrin and syndecan 4. This regulatory process is further accelerated on stiffened fibrillar matrices. It involves actin polymerization, actomyosin contraction, and the involvement of cytoplasmic proteins such as focal adhesion kinase, paxillin, Arp2/3, RhoA, and phosphoinositide 3-kinase. In addition, this immediate recognition and adhesion of fibroblasts to fibrillar fibronectin govern their migration speed, persistence, and proliferation over periods ranging from hours to weeks [71].

In addition to syndecan 4, syndecan 1 and 2 members can interact with integrins that modulate mechanotransduction when attached to the ECM. Syndecan 1 interacts with various integrins, including αVβ3, αVβ5, and α2β1. These interactions occur primarily through the HS chains attached to the extracellular domains of syndecan 1, which bind to ECM proteins such as fibronectin and collagen. Integrins bind to the same ECM proteins, forming a ternary complex with this PG [72,73].

**Figure 3:**
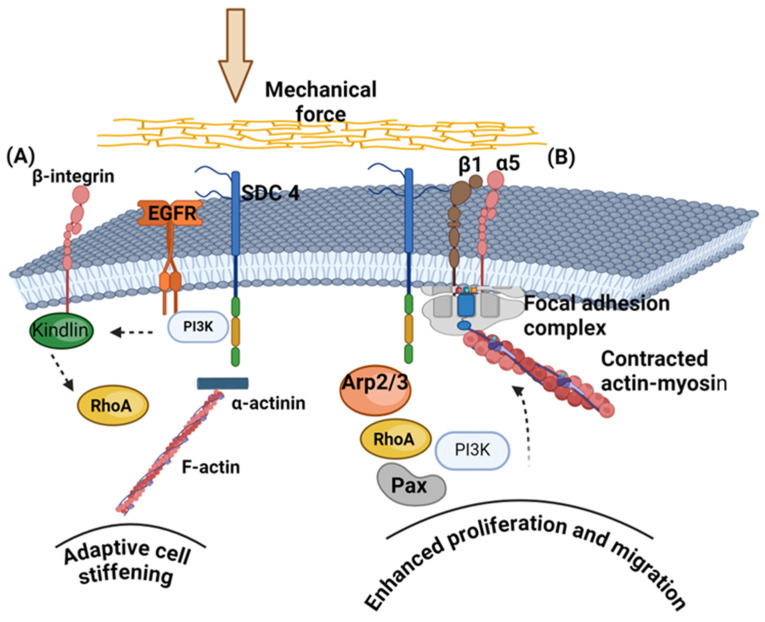
PG–integrin interactions in mechanotransduction (**A**) Force exerted on syndecan 4 activates the kindlin 2–β1 integrin–RhoA axis through PI3K and EGFR, causing a conformational change that helps form a syndecan 4–α-actinin–F-actin scaffold at adhesion sites. (**B**) α5β1 integrin and syndecan 4 bind to fibrillar fibronectin, initiating cell adhesion and activating signaling pathways involving Arp2/3, RhoA, paxillin, and PI3K. These pathways enhance adhesion to stiffer fibrillar FN through actin polymerization and myosin II-mediated contraction, which results in increased fibroblast proliferation and migration. Created in BioRender. Nikitovic, D. (2024) BioRender.com/j32l871 (accessed on 12 September 2024).

The role of PGs was investigated with bioengineering techniques [74]. For this aim, biomimetic PGs replicating natural PG nanoscale structures were synthesized to engineer the pericellular microenvironment to regulate cell mechanosensitive activities with minimal invasiveness. Articular cartilage was used as a model system due to its extensively studied pericellular matrix. The exclusive presence of type VI collagen, perlecan, biglycan, and aggrecan characterizes the pericellular matrix of cartilage. Aggrecan, a prominent cartilage PG, is mainly located in the pericellular matrix and shows a faster turnover than the bulk matrix. The synthesized biomimetic PGs mimic the nanostructure and negative charge of native aggrecan. Through evaluating their ability to modulate chondrocyte mechanotransduction, the authors found that PGs integrate into the native cartilage PCM via molecular adhesion interactions with aggrecan, enhancing pericellular matrix micromechanics and chondrocyte mechanotransduction, thus facilitating molecular engineering of cartilage [58]. Some examples of PG–integrin interactions in mechanotransduction are depicted in Figure 3.

#### 5.2.2. PGs and Cadherin Mechanotransduction Properties

Numerous cells within multicellular organisms engage with the ECM and establish communication with neighboring cells by forming cell–cell junctions, including adherens junctions, desmosomes, tight junctions, and gap junctions. Adherens junctions and desmosomes link the cytoskeletons of adjacent cells, facilitating the transmission of mechanical forces [75]. Cadherins are the major adhesion receptors within adherens junctions and desmosomes, mediating adhesion primarily through homotypic interactions. Classical cadherins, such as E-cadherin, are prevalent in adherens junctions, where they interact with α- and β-catenin to anchor the actin cytoskeleton of neighboring cells. In addition, the adherens junction includes p120 catenin, which regulates the clustering of cadherins. The coupling of adherens junctions to the actomyosin cytoskeleton enables these junctions to actively sense and transmit forces between neighboring cells. Analogous to the mechanosensitive protein talin in focal adhesions, α-catenin partially unfolds under high intracellular force, revealing a hidden binding site for vinculin. This event allows vinculin to strengthen the association between cadherin and actin [76,77].

Adherens junctions and focal adhesions can exhibit antagonistic or cooperative relationships, influenced by their connections to the actin cytoskeleton. Their mutual connection to the actin cytoskeleton results in a balanced distribution of forces between cell–cell and cell–ECM attachment sites. Thus, activation of integrin–matrix adhesions on fibronectin or collagen leads to increased tension at adhesion junctions and subsequent disruption of cell–cell contacts. Furthermore, the traction force exerted by cells on the ECM is directly correlated with the force generated between E-cadherin junctions in cell–cell pairing [78,79]. In mesenchymal and epithelial directional collective cell migration (DCCM), cadherin-mediated cell–cell adhesion maintains cohesion and facilitates mechanosignaling. P-cadherin (CDH3), often overexpressed in tumors, induces DCCM by activating the β-Pix–CDC42 polarity axis [80]. P-cadherin predicts intercellular tension levels and enhances stress anisotropy [81], guiding cell layers through plithotaxis. Tensile forces, essential for cell migration, are balanced by intercellular forces and stimulated by cadherin-based adhesion [80].

It has been suggested that cadherin-mediated mechanosignaling pathways may lead to changes in ECM organization, enhancing ECM–cell interactions to promote the effective generation of traction forces. P-cadherin has been demonstrated to play a pivotal role as a mediator of DCCM by initiating a novel signaling pathway. This pathway involves the upregulation of decorin, resulting in the orientation of collagen fibers in the direction of cell migration and the activation of β1 integrin and the β-Pix–CDC42 axis [82]. Intercellular tensions partly depend on the intercellular contact distances. For example, the Xenopus gastrula model demonstrated a range of intercellular contact distances from 10 to 1000 nm. The frequencies of contact widths characterize tissue-specific contact patterns, with knockdown of adhesion factors altering these patterns. A study by Barua et al. [83] highlights the significant role of the membrane PG syndecan 4 in all types of contacts, including narrow C-cadherin-mediated junctions. Additionally, syndecan 4, HA, paraxial protocadherin, and fibronectin influence contact widths. In a separate study, proline/arginine-rich end and leucine-rich protein (PRELP) was shown to activate EndMT and facilitate cell–cell adhesion of endothelial cells, presumably in a TGFβ-dependent manner [84].

#### 5.2.3. PG Cell–ECM Interactions

By regulating matrix stiffness, organizing the pericellular matrix, and modulating cell–ECM interactions, PGs can regulate mechanosensing. Modulation of matrix stiffness is a critical component of the mechanosensing machinery. For example, it is well established that PGs act as an osmotic sponge, trapping incompressible water and increasing tissue stiffness [85,86]. Interestingly, increased tissue stiffness increases PG expression, apparently creating a self-perpetuating cycle [87]. In mammographic density, normal breast tissue presents collagen behavior similar to stiffened cartilaginous collagen under pressure, with the increased alignment of collagen fibers adjacent to glands [88]. Syndecan 1 serves as a critical co-receptor for α2β1 integrin, facilitating adhesion to fibrillar type I collagen [89], and cell surface-expressed syndecan 1 has been demonstrated to maintain collagen alignment via an αvβ3 integrin bridge [90]. Recently, disruption of the role of syndecan 1 in maintaining collagen alignment via the αvβ3 bridge was shown to be essential for mediating mammographic density in an ex vivo model [91].

Involvement of the Hippo signaling pathway is a critical mechanism by which cells sense escalating mechanical force. The Hippo signaling pathway involves the transmission of mechanical signals via actin fibers across the cell cytoplasm, ultimately leading to the transcriptional activation of genes through YAP/TAZ-mediated activation of the DNA-binding protein TEAD [92]. Interestingly, a consensus TEAD-binding sequence is present in the syndecan 1 promoter, but not in syndecan 2 or syndecan 4, and indeed, TEAD has been shown to regulate syndecan 1 expression [93].

Another example of PGs modulating physicochemical properties is their interaction with the PIEZO receptors. PIEZO receptors are a class of mechanically activated ion channels in various cell types, including chondrocytes. These receptors play a crucial role in sensing mechanical forces, such as compression or stretching, and converting them into cellular responses [94]. PGs, such as aggrecan and perlecan, are abundant in cartilage ECM, involved in maintaining its structural integrity, and strongly remodeled in cartilage diseases [95]. PGs can help transmit mechanical forces from the ECM to cell surface receptors, including PIEZO receptors, with their negatively charged GAG chains. This transmission of mechanical forces can activate PIEZO channels, leading to intracellular signaling cascades and cellular responses. Studies suggest that PGs may act as mechanotransducers or facilitate the transmission of mechanical forces to PIEZO channels on chondrocyte membranes [94]. A recent study utilizing single-cell compression using atomic force microscopy (AFM) with finite element modeling (FEM) to identify the biophysical mechanisms of PIEZO-mediated calcium (Ca^2+^) signaling in chondrocytes determined that PIEZO1 and PIEZO2 are necessary for initiating Ca^2+^ signaling when cellular deformation reaches moderately high levels. However, at the highest strains, PIEZO1 functions independently of PIEZO2. In addition, this research suggests that PIEZO1-induced signaling is responsible for mechanical injury to chondrocytes caused by high membrane tension. The threshold may be altered by factors that affect membrane tension, such as cartilage hypoosmolarity resulting from PG loss [96]. The interaction between PIEZO receptors and PGs remains to be fully elucidated.

#### 5.2.4. PGs Affect the Response to Shear Stress Exerted by Blood Fluid on Endothelial Cells

The apical surface of endothelial cells is covered by a layer of glycans called the “GCX”, which consists mainly of PGs and HA. The endothelial GCX extends into the lumen of blood vessels and acts as a barrier between the vessel wall and the blood. This strategic positioning allows its various components, including major endothelial PGs such as glypican 1 and syndecan 1 and GAGs such as hyaluronic acid, to play a role in mechanosensation and mechanotransduction in response to stimuli such as fluid flow shear stress. Shear flow can be classified as either laminar or turbulent depending on the structure of the lumen. Uniaxial extensional (elongational) flow involves flow acceleration parallel to the vessel wall. In contrast, extensional stress is typically found in regions of sudden changes in fluid flow due to contraction or expansion. Laminar flow is beneficial to the vascular wall, aiding in anti-inflammation, anti-adhesion, and anti-thrombosis. In contrast, sustained turbulent flow can increase endothelial permeability and promote pro-inflammatory signaling, such as the activation of nuclear factor κB (NF-κB) and adhesion molecules, leading to lesion formation [97]. Fluid shear stress (FSS) influences tissue homeostasis in blood vessels, the heart, airways, and the urinary bladder. In the vascular wall, high FSS induces anti-inflammatory effects, such as the activation of Klf2/4 and endothelial nitric oxide synthase (eNOS). Conversely, turbulent, oscillatory, and low FSS induce pro-inflammatory responses. In the circulatory system, FSS is generated by heart contractions and is determined by wall shear rate and blood viscosity [98]. In particular, to coordinate the myriad processes triggered by physical forces, the GCX interacts with numerous membrane and cytoskeletal proteins, activating specific signaling pathways that lead to different responses of endothelial cells and blood vessels to mechanical forces [99,100].

Hox et al. proposed that the syndecan family of GCX core proteins, particularly syndecan 1 in endothelial cells, links the GCX to the cytoskeleton. This link plays an important role in allowing mechanically induced morphological changes in endothelial cells [85]. Syndecan 1 has been shown to rapidly interact with Src and calmodulin in response to shear stress, facilitating actin alignment [101]. In a separate study, syndecan 1 was shown to play a critical role in fluid shear mechanotransduction, reorganizing the actin cytoskeleton to align with the flow, and its absence resulted in dysregulated flow and a pro-inflammatory phenotype of endothelial cells. Specifically, this study discovered that knocking out the syndecan 1 gene resulted in the loss of key initial responses in endothelial cells when exposed to shear stress. These responses included the activation of Akt, the establishment of a spatial gradient in paxillin phosphorylation, and the triggering of RhoA [102].

The cytoplasmic domain of syndecan 1 is associated with the actin cytoskeleton through two conserved regions (C1 and C2) and one variable region (V) [103]. Shear stress enhances the association between syndecan 1, actin, Src, calmodulin, and myosin IIb, forming a GCX–cytoskeletal network that helps endothelial cells withstand and adapt to shear stress [99,101].

A recent computational study showed that a significant mode of flow shear stress transmission in endothelial cells involves a scissor-like movement of the syndecan 4 cytoplasmic domain. The results suggest that the force transmitted into the cytoskeleton is in the range of 10 to 100 pN and that the primary role of the GAG chains of a GCX component is to protect the core proteins from significant conformational changes, thus preserving the functionality of the endothelium [104]. Furthermore, syndecan 4 at the basal membrane has been shown to be involved in the transduction of shear forces into biochemical signals [105]. Therefore, the tension generated in syndecan 4 by apical shear induces a conformational change in the cytoplasmic domain, the variable region of which is essential for the mechanical adaptation to force, facilitating the assembly of a syndecan 4–α-actinin–F-actin molecular scaffold, promoting actin polymerization and altering the cytoskeletal structure [26,105]. Actin polymerization leads to a comprehensive cellular remodeling response by establishing an actin cytoskeletal network and initiating the formation and migration of focal adhesions [106]. These changes induce morphological transformations in cells and tissues, facilitating endothelial cell actions, such as elongation, alignment, polarity, migration, and proliferation [26]. Moreover, a shift in the structure of the syndecan 4 cytoplasmic region [105,107] occurs upon exposure to tension. The cytoplasmic segment of syndecan 4 exhibits distinct Rho conformational patterns that facilitate its engagement with Rho inhibitors and GTPases. This distinctive feature enables syndecan 4 to directly regulate the Rho pathway as a modulator [105].

Atherosclerotic plaque tends to form in areas of disturbed flow where endothelial cells are poorly aligned, while sustained laminar flow promotes proper cell alignment and resistance to atherosclerosis. Removal of syndecan 4 significantly increased plaque burden in hypercholesterolemic mice, even in areas normally resistant to plaque. Endothelial cells from these mice were poorly aligned with the direction of flow. Similarly, depletion of syndecan 4 in human endothelial cells inhibited flow-induced alignment, which was restored by re-expressing syndecan 4. Although flow activation of VEGF receptor 2 and NF-κB was unaffected, syndecan 4-depleted cells showed increased pro-inflammatory NF-κB and decreased anti-inflammatory KLF2 and KLF4 under laminar flow. Therefore, syndecan 4 is crucial for sensing flow direction, promoting cell alignment, and preventing atherosclerosis [108].

**Figure 4:**
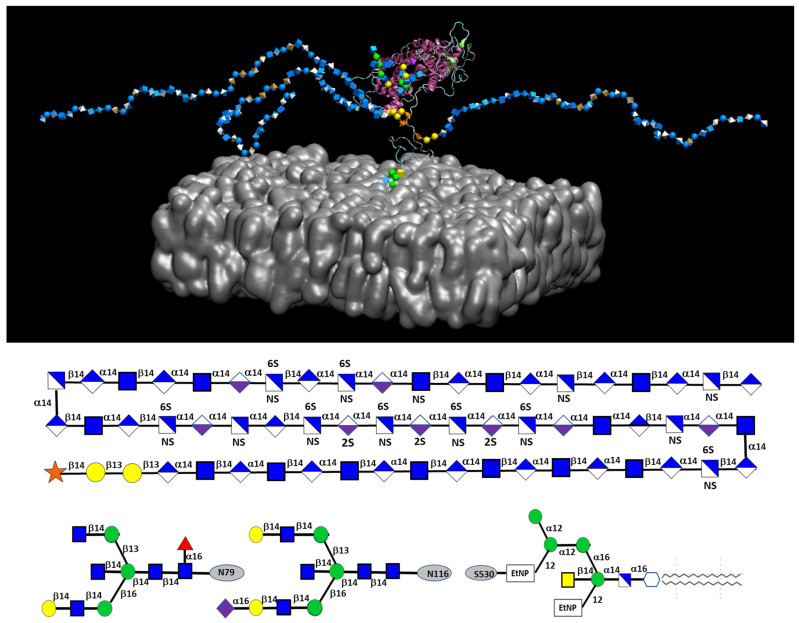
Snapshot of the glypican 1 system comprising: glypican 1 protein, N-glycans, GPI-anchor, and three heparan sulfate chains (degree of polymerization: 30) linked to the protein and membrane (adapted from Dong et al., 2021 [109]). Color displayed in the monosaccharide units follows the SNFG recommendations. Perez, Serge; Nikitovic, Dragana (2024). Glypican 1 system. figshare. Figure: https://doi.org/10.6084/m9.figshare.26983060.v1 (accessed on 12 September 2024). CC by 4.0.

Glypican 1, a core GCX protein, is involved in transferring shear force into endothelial biochemical signaling [99]. Figure 4 displays a snapshot of the glypican 1 system, as established by molecular dynamic simulations [109].

**Figure 5:**
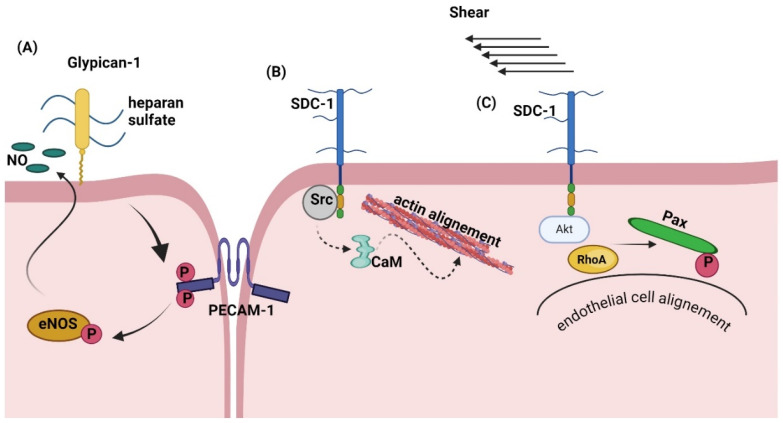
PGs transduce shear stress to regulate endothelial cells’ functions. (**A**) Glypican 1 senses shear force to initiate the PECAM1–eNOS axis and increase NO production. (**B**) In response to shear stress, syndecan 1 interacts with Src and calmodulin (CaM), enhancing actin alignment and endothelial cell cytoskeleton reorganization. (**C**) Syndecan 1 perpetrates critical initial responses to shear stress, including Akt activation, creating paxillin phosphorylation gradients, and RhoA activation, which results in aligning the actin cytoskeleton with the flow. Created in BioRender. Nikitovic, D. (2024). BioRender.com/i78n493 (accessed on 12 September 2024).

Atomic force microscopy was applied to glypican 1 on human endothelial cells, and nitric oxide production was determined. Glypican 1 stimulation increased nitric oxide production, whereas PECAM-1 stimulation did not, although PECAM-1 was necessary for the effect of glypican 1. Glypican 1 knockout mice showed impaired flow-induced endothelial nitric oxide synthase (eNOS) phosphorylation without changes in PECAM-1 expression. The study revealed a cooperative mechanism whereby glypican 1 senses flow and activates PECAM-1, leading to eNOS phosphorylation and nitric oxide production [110]. In addition, another recent study showed that endothelial cells cultured on stiff polyacrylamide gels had reduced GCX expression and increased endothelial dysfunction compared to cells on softer gels. Glypican 1 expression was significantly inhibited on stiff gels, resulting in increased inflammation and monocyte adhesion, and inhibited nitric oxide expression. Gene silencing or overexpression of glypican 1 confirmed its protective role against stiffness-induced endothelial dysfunction. Older mice with naturally stiffer arteries showed reduced glypican 1 expression and more significant endothelial dysfunction, a condition exacerbated by glypican 1 deletion in young but not old knockout mice [111]. The role of PGs in shear stress transmission is shown in Figure 5.

## 6. GAG/PG-Mediated Mechanosensing in Cancer

### 6.1. Tumor Microenvironment and ECM Remodeling

The cancer microenvironment is an extensively studied system involving multiple players: cellular components (i.e., stromal cells, fibroblasts, immune cells, endothelial cells, and tumor cells), ECM molecules, and soluble components. Interactions between these and cancer cells modulate tumor cell growth, immune responses, angiogenesis, and metastasis [112]. As for the ECM molecules, changes in their interactions, density, and cross-linking of ECM fibers lead to irregular matrix stiffness and cancer development [113,114]. Collagen accumulation by cancer-associated fibroblasts in the tumor ECM leads to fibrosis and malignant transformation [115,116]. In addition, the synthesis, secretion, localization, accumulation, and interaction of PGs and GAGs with other ECM molecules and growth factors [117,118] varies between cancer types. It modulates cell function (proliferation, migration, adhesion) and tumor differentiation [119,120]. Several studies indicate the potential of GAGs and PGs as prognostic or diagnostic markers due to variations in their quality and quantity [121]. Interestingly, GAG effects seem to be highly dependent on the tumor type, and further studies are needed to uncover their complexity [122]. PGs and HA (a non-sulfated GAG, not covalently linked to PGs) contribute to cancer-associated modulation of the immune response [119,123,124]. In addition, ECM remodeling is characterized by increased expression of enzymes such as MMPs, lysyl oxidase (LOX), lysyl oxidase-like proteins (LOXLs), WNT1-inducible signaling pathway proteins (WISPs), and others [125]. These enzymes can control tissue stiffness and cell–matrix interactions [126]. More specifically, MMPs have been associated with the degree of tumor malignancy in many epithelial cancers, such as lung, breast, and pancreatic cancers, due to their remodeling function [127,128].

### 6.2. Dysregulated Mechanosensing in Cancer Progression

Mechanosensing and related pathways in cancer involve a plethora of molecules and different mechanisms [129]. Cancer initiation and metastasis depend on irregular mechanical changes, such as structural, morphological, and stiffness changes, in both cells and the ECM, as well as unique genetic and biochemical factors associated with tumor development [130,131,132]. The disease stage correlates with tumor stiffness [133]. Cancer development modulates cell–cell and cell–ECM interactions and cytoskeletal remodeling, leading to tumor cell formation [134] and initiation of tumor cell migration from the primary site, leading to intravasation. Circulating tumor cells resist the mechanical forces in the bloodstream to survive and migrate to the secondary organ, where they use forces and undergo morphological changes to escape the vasculature and enter the ECM of the secondary organ [131,135,136].

Therefore, changes in the ECM during cancer development and metastasis result in different physical forces in tissues that modulate their mechanobiology. Animal experiments have shown differences in tissue mechanical properties such as stiffness, fibrillar collagen orientation, and cross-linking density [137,138]. Key mechanosensing signaling molecules such as focal adhesion kinase (FAK), cadherins, integrins, and syndecans were shown to be activated [90,139,140], resulting in modulation of metastatic dissemination, matrix-independent survival and chemotherapeutic resistance of different cancer types [141,142]. Furthermore, increased subcellular localization and activation of YAP in the nucleus increased cancer cell migration [143,144]. Changes in the ECM in cancer can also modulate the behavior of adjacent fibroblasts and endothelial and immune cells, which also affects metastatic potential [145]. Cancer-associated fibroblasts can stimulate fibroplasia and the assembly of matrix cross-linking enzymes that regulate ECM changes [146]. The secretion of several chemokines also protects tumor cells from cytotoxic T cells of the immune system and promotes their growth [147]. The elucidation of mechanical tissue and cell homeostasis in cancer microenvironments is essential for understanding and manipulating cancer responses.

The tumor environment consists of different cell types, including fibroblasts, endothelial cells, immune cells, and the ECM. This environment results from remodeling the physiological ECM, which the pathological tumor ECM replaces. In addition, ECM degradation releases growth factors, cytokines, and mitogens that promote tumor proliferation, invasion, and metastasis [148].

#### 6.2.1. ECM Stiffness and Desmoplasia

ECM stiffness [149], a rigid ECM consisting mainly of collagen, fibronectin, PGs, and HA [150], and the presence of desmoplasia, the accumulation of dense fibrosis around the tumor, characterize the tumor environment and are correlated with cancer development.

Desmoplasia formation leads to increased MMPs in several types of cancers, causing changes in tissue homeostasis [151]. In addition, variations in the expression of collagen types I, III [152], and IV [153] modulate tissue homeostasis associated with tumor desmoplasia. In addition, desmoplasia facilitates cancer resistance to therapy and immune escape [154]. ECM stiffness is also associated with the upregulation of collagen levels and cross-linking: the latter is mainly mediated by LOX and LOXL enzymes [155]. Permanent cross-linking occurs via enzymatic LOX, transglutaminase (Tg)] or non-enzymatic reactions (advanced glycosylation end products (AGEs)) [156]. LOX covalently cross-links lysine and hydroxylysine regions of elastin and type I collagen chains, resulting in increased cell motility and adhesion capacities through induction of the FAK–Src signaling axis [157]. LOX also increases YAP’s nuclear transfer by forming integrin-mediated FAK complexes [158]. High expression of LOX has been reported in several cancers, including oral [159] and pancreatic cancers [160]. The combination of LOX inhibitors with other chemotherapeutic or immunotherapeutic agents has been shown to modulate collagen cross-linking. Such a combination improves CD8+ T cell accumulation and overcomes drug resistance and immune evasion in xenograft models of triple-negative breast cancer (TNBC) [161,162].

Moreover, Park et al. suggest that matrix stiffness controls the expression of PD-L1, an immune suppressor molecule, via YAP activation, ultimately contributing to cell proliferation in lung adenocarcinoma [163]. There is overexpression of receptors for AGEs (RAGEs) in cancer cells [164], and their interaction leads to apoptosis and increased angiogenesis in the tumor microenvironment [165]. RAGE-expressing fibroblasts have been shown to mediate the conversion of naïve fibroblasts to cancer-associated fibroblasts upon ligand interactions and integrin-mediated upregulation of mechanoresponsive genes [166], highlighting the importance of their expression in the surrounding tumor tissue. Overall, AGE mediates ECM stiffening, promotes epithelial cell invasion, and decreases prostate cancer survival [167].

Furthermore, the tumor environment is characterized by post-translational modifications of ECM proteins, such as hydroxylation, phosphorylation, N- and O-glycosylation, acetylation, ubiquitylation, sumoylation, and methylation [168]. Other modulated ECM molecules affecting tissue stiffness are elastin (27), HA [169,170,171], and fibronectin [172]. All these promote a tumor stroma with high interstitial pressure, favoring both tumor proliferation and metastasis [173].

**Figure 6:**
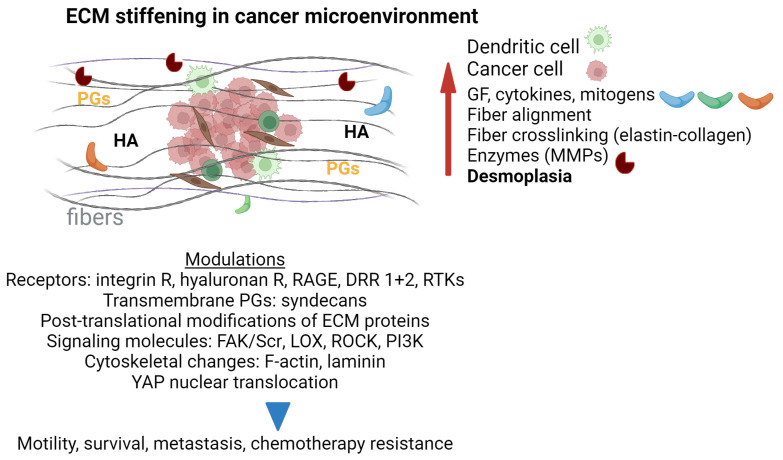
Parameters of ECM stiffening in the cancer microenvironment. During cancer development, ECM stiffening and desmoplasia occur. ECM fiber alignment and fiber cross-linking together with the secretion of growth factors (GFs), cytokines, mitogens, and enzymes (MMPs) by cancer-associated fibroblasts (brown cells) and immune cells (green cells) lead to modulation of mechanosensing and mechanotransduction pathways (receptors, signaling molecules, and cytoskeletal proteins). Finally, all the above lead to changes in cancer cell functions, such as motility, survival, metastasis, and chemotherapy resistance. Created in BioRender. Nikitovic, D. (2024). BioRender.com/k83x155 (accessed on 12 September 2024).

Tumors with high stiffness and desmoplasia, such as breast, pancreatic, and lung cancer, are characterized by chronic inflammation, fibroblast expansion, related activation in the cancer-associated fibroblast phenotype, and increased angiogenesis [173]. Studies have also shown that both collagen receptors and other ECM molecules, including discoidin domain receptor family (DDR1 and DDR2), integrins, HA receptors (CD44 (cluster of differentiation 44), RHAMM (hyaluronan-mediated motility receptor), Toll-like receptors (TLRs)), and fibronectin receptors (a5b1, avb3, a4b1) are involved in the mechanotransduction of the stiff matrix [173]. See Figure 6.

#### 6.2.2. Fiber Alignment

Fiber orientation modulates tissue stiffness and has been associated with adverse cancer prognosis [174,175]. External fibril ECM forces activate cell stress fibers, which can also be activated by interaction with integrins, Rho/ROCK induction, and phosphorylation of myosin light chains [176]. Tumor-associated fibroblasts and macrophages are thought to be involved in collagen orientation [177,178]. For example, receptors (syndecan 1) found in such fibroblasts have been shown to induce directional persistent migration in breast cancer cells [179]. Macrophages in the tumor environment also secrete proteases that aid in the degradation and synthesis of collagen fibrils [180]. Elucidating the mechanisms of fiber orientation changes at a distance from the tumor or through ECM remodeling would help to understand tumor–stroma interactions and systemically induced effects.

#### 6.2.3. Cancer Cell Movement

Cancer cells can migrate through the ECM within a tissue or between tissues. This function is thought to be achieved by either a protease-dependent or protease-independent mode [181]. Cells move through small or large spaces by changing shape and reducing their adhesion capacity to the surrounding ECM obstacles (fibers) using protease-independent migration [182,183]. Different patterns formed by fibrils and fibril stiffness affect cell mechanosensitivity and cancer cells’ migration and adhesion mechanisms to metastasize [184].

Two mechanisms play a critical role in the movement of cancer cells in tissues with different ECM stiffness. The first pathway involves activation of the PI3K signaling pathway by the cells, which enables movement in ECMs with high interfibrillar spacing, and the second pathway involves activation of the ROCK signaling pathway, which inhibits PI3K signaling when moving through spaces with low interfibrillar spacing [185]. Cancer cells, in contrast to normal cells, have been shown to climb walls on substrates with microgroove structures and are sensitive to changes in angle [184,186]. The cell actin cytoskeleton’s dynamics and interactions are critical for cancer cell migration [187].

When cells cannot migrate through the ECM, protease-dependent migration mechanisms are activated, and ECM-degrading enzymes such as serine proteases, MMPs, and cathepsins are secreted. Studies have shown that protease inhibition does not attenuate tumor cell migration, even in high-density ECMs [188]. In particular, a study using breast cancer and fibrosarcoma cells showed that MMP inhibitors increased the softening of nuclei (nuclei are relatively rigid) by lamin A/C phosphorylation [189].

#### 6.2.4. Elasticity, Viscoelasticity, and Plasticity

Elasticity in tumor tissue also modulates cancer progression [184]. Integrins on cell membranes can sense tissue stiffness via complex downstream mechanotransductive signaling pathways [190]. Depending on the tissue, cancer cells have been shown to predominantly migrate towards areas of increasing stiffness, such as breast cancer cells. Some types, such as glioblastomas, migrate towards softer regions [184,191].

The ROCK isoforms ROCK1 and ROCK2 in breast cancer cells modulated activation of myosin regulatory light chain (MRLC) and cofilin, which changed F-actin depolymerization and led to cytoskeletal remodeling [192]. In addition, talin inhibition in breast cancer cells resulted in migration to an intermediate-stiffness region, highlighting its role in cell migration sensing. Intracellular YAP expression also induces actomyosin contractility in fibroblasts, accumulating fibrils at the growth front and enabling cancer cell migration [193]. Tumor macrophages were found to regulate TGFβ expression levels, thereby promoting malignant transformation [194]. HA also regulated matrix stiffness and tumor invasion in pancreatic cancer [195] and brain tumors [196].

The ECM exhibits both elastic and non-elastic properties, such as viscoelasticity and plasticity [197,198], due to the different quality of cross-linking between its components. Viscoelastic matrices can reach purely viscous or purely elastic states depending on different external forces or loads that are applied or removed occasionally. Tumor cells in viscoelastic environments increase their vimentin levels and decrease cytokeratin levels, thereby increasing their migration potential through the epithelial-to-mesenchymal transition [199]. Deng et al. constructed a matrix with tunable viscoelasticity. They showed that MG63 osteosarcoma cells exhibited viscoelasticity-dependent behavior even discretely compared to normal cells/mesenchymal stem cells cultured in the viscoelastic matrix [200].

In addition, viscoelasticity modulates stress fiber formation and increases nuclear translocation of YAP, which positively correlates with tumor metastasis and chemoresistance [201]. Mechanical plasticity is a component of viscoelasticity in which the matrix undergoes irreversible deformations. The matrix can be considered viscoplastic when it exhibits both plastic and viscoelastic properties. Cancer cells can outgrow different plasticity matrices by expressing different adhesion and matrix remodeling-related genes [197].

### 6.3. Impact of GAGs and PGs on Cancer Cell Behavior

GAGs and PGs are components of the ECM that aid mechanosensing and the downstream signaling that affects tumor cell function [129,202]. PGs in the ECM can bind to a wide range of matrix proteins, such as collagen, fibronectin, and laminin, modulating tissue stiffness [203,204] and tumor pathology. In addition, GAGs can exert mechanical pressure that causes curvature of the cell plasma membrane, leading to the formation of cell surface structures such as microvilli, filopodia, and lamellipodia that aid tumor cell function; migration, adhesion, invasion, and drug resistance [129].

#### 6.3.1. Syndecans

The HS-linked PG family of syndecans (1 to4) have been documented to alter their expression levels in cancer and to affect tumor cell function in different ways depending on the type of cancer. More importantly, the core protein and the HS chains of cell surface or shed syndecans modulate cancer progression [205,206]. The interaction of syndecans with growth factors and their receptors also defines their biological role in cancer [61]. They were found to be involved in the formation of RTK/GF signaling complexes, altering ligand binding and modulating downstream RTK signaling, thereby affecting cell growth, survival, adhesion and metastasis [61]. More specifically, syndecan 1, upon association with ECM content, promotes the binding of its ectodomain to integrin avb3, resulting in avb3 activation and formation of a standard “docking point” for IGF-IR activation of downstream signaling [61]. The syndecan 2 protein core binds to both TGFβ and the type III TGFβ receptor (TβRIII), betaglycan, facilitating ligand binding, receptor TGFRI activation, and initiation of downstream Smad signaling [61]. Furthermore, loss of syndecan 1 expression in the majority of epithelial tumors like cervical, lung, head and neck, squamous cell, and esophageal cancers is associated with tumor development and reduced patient survival [129]. However, increased syndecan 1 expression in breast, pancreatic, ovarian, thyroid, and endometrial tumors is associated positively with tumor development [207]. Another action of syndecan 1 and other HSPGs is the formation of fibronectin and collagen 1 fibers, which enable cancer cells to migrate [139].

Syndecan 2 directly mediates IGFI-induced extracellular signal-regulated kinase 1/2 (ERK1/2) activation, recruits ezrin, contributes to actin polymerization and ezrin–actin membrane localization, and ultimately facilitates the progression of IGFI-dependent fibrosarcoma cell migration [208]. Similarly, in fibrosarcoma cells, syndecan 2 regulates TGFβ2 transcriptional regulation via Smad signaling to facilitate fibrosarcoma cell adhesion [209]. Regarding melanoma progression, FGF-2 modulates melanoma migration capacity through a syndecan 4-dependent mechanism [210]. Syndecan 4 can transduce mechanical force in fibroblasts and pancreatic stellate cells [66,211]. When pulsed forces were applied to magnetic beads coated with antibodies against the core protein of syndecan 4, there was a mechanical stiffening response similar to the stiffening response upon force used on integrins [66,211]. The application of force to syndecan 4 resulted in a modified cytoplasmic domain and increased its binding to alpha-actin, a scaffolding protein present in cell–ECM adhesions, providing a mechanism for tissue stiffening [66]. In addition, mechanical activation of syndecan 4 promoted the assembly of focal adhesions and stress fibers in fibroblasts [212,213]. Other studies described the role of syndecan 4 in mechanosignaling pathways of melanoma, glioblastoma, and osteosarcoma cells [214,215,216].

The human genome encodes a single heparanase, which cleaves HS chains into biologically active oligosaccharides. This process can impact the distribution of Hep-binding ligands, such as growth factors, affecting gene expression and cell motility. Elevated heparanase levels are observed in several tumor types and are frequently associated with poor prognosis (reviewed in [10]). Cleavage of HS chains can affect various mechanical cues, including those regulating tumor cell migration, adhesion, invasion, and drug resistance [61,129].

#### 6.3.2. Agrin

Agrin is another HSPG that is secreted into the ECM or localized to the cell membrane [217]. Elevated levels of agrin have been associated with tumor aggressiveness in oral squamous cell carcinoma [218] and hepatocellular carcinoma [219]. In hepatocellular carcinoma, agrin mediated the Lrp4-Musk signaling pathway and increased the accumulation of cell–ECM adhesions [219]. Experiments using 2D polyacrylamide hydrogels showed that agrin levels were higher in cells on stiff 2D collagen-coated polyacrylamide hydrogels than in those cultured on soft 2D polyacrylamide hydrogels [220]. In the same study, loss of agrin inhibited YAP nuclear translocation on stiff ECM, whereas exogenous addition of agrin induced YAP nuclear translocation [220], demonstrating the involvement of agrin in mechanically induced ECM stiffness in hepatocellular carcinoma cells.

#### 6.3.3. Serglycin

Serglycin, an intracellular PG and secreted by cancer cells, linked to either Hep or CS chains [221], can bind to cell surface receptors [222,223] and modulate cell function. Studies found overexpression of this PG in hematological malignancies, gliomas, and tumors of the breast, prostate, lung, and liver [224,225]. The role of serglycin in cancer mechanotransduction is still under investigation, and initial investigations have demonstrated its involvement in FAK signaling and YAP expression in breast cancer cells [226,227]. Serglycin was found to upregulate YAP through integrin α5/FAK/CREB signaling, resulting in increased histone deacetylase 2 (HDAC2) expression, which modulates stemness and chemoresistance in breast cancer cells [226].

#### 6.3.4. Small Leucine-Rich PGs (SLRPs)

Members of the SLRP family (mainly biglycan, lumican, and decorin) modulate cancer cell functions such as proliferation, migration, adhesion, and invasion in several cancer types (reviewed in [114]). Biglycan and decorin regulate collagen fibril structure and fiber orientation, affecting matrix assembly and tissue stiffness [129,228,229,230]. The effects of decorin on cancer are conflicting and require further investigation.

Biglycan stimulates the formation of a denser collagen architecture and increased tissue stiffness, which leads to the upregulation of β1-integrin expression and promotes melanoma cell invasion [204]. The same SLRP also increased tumor cell invasion and gastric cancer metastasis through FAK phosphorylation at Tyr576/577, Tyr925, and Tyr397 [231]. Biglycan-deficient breast cancer stem cells showed reduced metabolism and decreased ability to form tumor spheroids [232]. In contrast, biglycan was shown to regulate desmoplasia in colorectal cancer by inhibiting migration and invasion of these tumor cells in 2D and 3D co-culture systems [233]. Biglycan can stimulate the growth of mesenchymal-derived tumor cells: such mechanisms have been investigated, and biglycan was found to modulate the insulin-like growth factor receptor I (IGF-IR) and Wnt/β-catenin signaling cascade, thereby promoting osteosarcoma cell proliferation [234].

Lumican represses invadopodia and lamellipodia formation in prostate cells through reduced rearrangement of ZO-1, keratin 8/18, integrin β1, and membrane type I matrix metalloproteinase (MT1-MMP) [235]. Similarly, lumican reduced invadopodia formation in melanoma cells [236]. In addition, this SLRP reduced the migratory ability of melanoma by binding the core protein to α2β1 integrin [237]. Lumican has been shown to reduce MMP-releasing invadopodia to inhibit melanoma lung metastasis in vivo [238] and to decrease the expression of MMPs to inhibit breast cancer migration and invasion [239]. It is well established that lumican controls tumor cell proliferation in a cancer type-dependent manner [213,240]. Cancer-associated fibroblasts in gastric cancer express lumican, which promotes the activation of the integrin β1–FAK signaling pathway, resulting in increased cancer cell proliferation and tumor progression [241]. Further studies have demonstrated the role of lumican in chondrosarcoma [242] and osteosarcoma proliferation signaling modulated by growth factors [243,244].

#### 6.3.5. Hyaluronan (HA)

HA a non-sulfated GAG, is overexpressed in most cancers, and its role in cancer cell function and development has been extensively studied [245,246,247,248]. Elevated HA levels have been associated with glioma tissue stiffness [249]. HA accumulation has also contributed to solid mechanical stress by increasing interstitial pressure through water retention in tumor tissue [250]. Studies in glioblastoma multiforme (GBM), an invasive brain tumor associated with abnormal HA secretion, tissue stiffening, and CD44 overexpression, showed upregulation of the transcripts related to HA/CD44 adhesion [251]. In addition, adhesion and migration rates are dependent on HA hydrogel stiffness, suggesting that CD44-based signaling is fundamentally mechanosensitive [251]. In addition, findings show that CD44 transduces HA-based stiffness signals before integrin-based adhesion maturation and facilitates invasion [251]. Increased expression of GAGs, HA, PGs, and fibrous proteins leads to stiffening of the brain ECM, which alters cellular signaling activity and function. Several mechanosensing signaling pathways have been described, such as Hippo/YAP, CD44, and actin skeleton signaling, which remodel the cytoskeleton and affect cellular properties such as cell–ECM interactions, cell proliferation, and migration of GBM cells [252]. Interestingly, Pranda et al. experimented with the effects of HA cross-linking on metastatic breast tumor cell migration and incorporation into human brain endothelium, a critical part of the blood–brain barrier (BBB) [253]. Metastatic breast tumor cell migration velocity, diffusion coefficient, spreading area, and aspect ratio increased with decreasing HA cross-linking, a mechanosensing trend that correlated with tumor cell actin organization, but not CD44 expression [253]. In contrast, incorporating breast tumor cells into endothelial monolayers was independent of HA cross-linking density, suggesting that changes in HA cross-linking density only affect tumor cells after they have left the vasculature [253].

#### 6.3.6. Other Chondroitin Sulfate PGs (CSPGs)

Changes in the expression of CSPGs are associated with pathological conditions such as cancer by modulating cellular functions and responses, including proliferation, apoptosis, migration, adhesion, invasion, and ECM assembly, through their highly negatively charged CS/DS side chains [254]. Versican is one of the major CSPGs highly expressed in cancer cell types such as osteosarcoma, testicular tumors, breast, pancreatic and colon cancer [255,256]. CS sulfation is essential for growth factor-mediated signaling and cancer progression. The CS chain and growth factors interact to affect the storage of growth factors and their release into the ECM, ultimately affecting cell signaling. Interestingly, the specificity of CS interactions with their ligands differs from that of HS chains. The different subtypes of CS chains have also shown to be critical in cancer development [255,257,258]. For example, the sulfation pattern in cancer progression has been correlated with selectin expression and MMP modulation, essential factors in ECM remodeling and tissue stiffness [259]. Furthermore, expression of CSPGs in melanoma cells increased integrin functions and activation of the ERK1/2 pathway, stimulating cell growth and motility [260]. The different structures of CS chains on versican and decorin have also been identified in pancreatic, rectal, and gastric carcinomas, where the expression composed predominantly of 6-O-sulfated and non-sulfated disaccharide units was increased [254,255]. Serglycin, which contains CS-A chains composed mainly of 4-sulfated disaccharides, is secreted by multiple myeloma cells and is involved in their immunoresistance [223]. Changes in CS metabolism regulated by their specific degradation enzymes are also crucial for cell malignant ECM remodeling and cancer progression [261].

## 7. GAG/PG-Mediated Mechanosensing in Inflammation and Fibrosis

### 7.1. ECM Remodeling during Inflammation

In the inflammatory response, fragments of the ECM act as activators of TLRs, mainly TLR4 and TLR2. Examples of molecules that are enzymatically processed in inflammation are the tenascin C isoform, the SLRP biglycan, fibronectin, and HS and HA [262]. The interaction of ECM components with TLR4 and TLR2 is a subject of intense research. For example, tenascin C, expressed at low levels in normal synovia, is upregulated in inflamed tissue. Therefore, tenascin C deposited in inflamed synovial fluid and cartilage from rheumatoid arthritis patients interacts with TLR4 expressed on macrophages and synovial fibroblasts, inducing the secretion of pro-inflammatory cytokines [263]. Notably, tenascin C-deficient animal models exposed to a TLR2 agonist do not develop synovitis [263]. Resident fibroblasts and macrophages in inflamed conditions have high production of pro-inflammatory cytokines (e.g., IL-6, TNF, CXCL8), and tenascin C maintains the inflammatory response in the joint.

Non-ECM-bound biglycan binds to TLR2 and TLR4 expressed on macrophages. This binding induces upregulation of CXCL2 and TNF on macrophages, promoting macrophage infiltration and perpetuating the inflammatory response [264]. Conversely, ECM-bound biglycan sequesters cytokines (e.g., TGFβ), thereby controlling local concentrations of this anti-inflammatory cytokine. Under normal conditions, biglycan is sequestered in the ECM as a structurally linked molecule with collagen types I, II, III, and VI, elastin, and TGFβ. However, in inflammation and ECM remodeling, biglycan is rapidly released by the action of various proteinases [265]. In pathologies associated with obesity and atherosclerosis, biglycan expression is dysregulated. In addition, biglycan is highly expressed in cancer, enabling tumor growth, invasion, and metastasis [266].

HA is also involved in TLR2 and TLR4 activation. Unlike other glycosaminoglycans, HA is synthesized at the inner side of the plasma membrane by HA synthetases and secreted into the extracellular milieu [267]. Various epithelial cells, endothelial cells, and fibroblasts produce HA, which contributes to forming the pericellular matrix by binding to its cellular receptor CD44 [268]. HA has a high affinity for water, is an essential structural component of the tissue interstitium, and circulates in body fluids. Free oxygen radicals rapidly degrade it and are enzymatically degraded by hyaluronidases [268]. Once cleared from tissues, it circulates in the lymph or is bound by receptor-mediated endocytosis.

**Figure 7:**
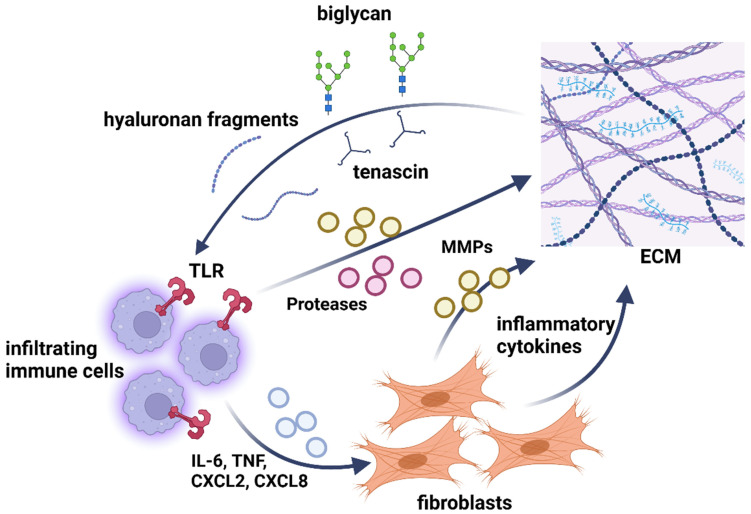
Inflammatory network of the ECM components. Infiltrating immune cells that arrive at the inflamed tissue synthesize and secrete cytokines, chemokines (CXCL, CXC chemokine ligand, IL-6, interleukin 6, TNF, tumor necrosis factor), proteases, and MMPs. All these molecules activate resident cells in the interstitium, alter ECM synthesis and/or inflict cleavage of ECM components, releasing biglycan, tenascin, and generating HA fragments. These ECM components enhance the inflammatory response by modulating immune cell chemotaxis, activation, differentiation or survival, perpetuating the inflammatory response by activating Toll-like receptor (TLR) 2 and 4.

Enzymatic degradation of HA produces low-molecular-weight HA fragments with pro-inflammatory effects [119]. One of the first published studies almost 20 years ago identified these low-molecular-weight HA fragments in bronchial lavage fluid after acute lung injury [269]. These fragments increase the pro-inflammatory milieu in tissues [270], interact with TLR2 and TLR4 expressed by immune cells, and stimulate further pro-inflammatory cytokines and chemokines [271]. In addition, this pro-inflammatory microenvironment increases interactions between antigen-presenting cells and T cells [272,273], prolonging the inflammatory response. All these extracellular molecules are endogenous danger signals within the inflammatory process in tissues. Their effect is to activate resident immune cells, even if pathogens are no longer present. The ECM fragments are therefore a mechanosensing tool that provides a platform for detecting injury caused by traumatic physical tissue destruction and microbial invasion, where pathogen-associated molecular patterns (PAMPs) are associated. This role is further supported by the changes in the mechanical properties of the ECM and the resulting mechanical cues under inflammation [2,3]. Figure 7 represents the network in which ECM degradation maintains the inflammatory pattern of a tissue.

### 7.2. GAG/PG Involvement in Immune Cell Recruitment and Activation

The ECM is an intricate network of macromolecules in which PGs, GAGs, and fibrous proteins are the major structural components in all tissues and organs. In addition to its role as a mechanical scaffold that supports cells, the ECM mediates key events for tissue homeostasis. Various deregulations of ECM deposition can further induce changes in the cell microenvironment that can trigger the onset and progression of multiple pathologies, including cancer. The conversion of the ECM from a healthy to a remodeling state is the main characteristic of tissue response to physical injury, inflammatory status, fibrosis, and neoplasia. The ECM changes its structure and physical and chemical properties to cope with new biomechanical and biochemical events imposed by the developed pathology. PGs and GAG modification support the regulatory processes of matrix organization within tissue healing after various inflicted injuries [274].

The endothelial cell is surrounded by a negatively charged gel-like layer called the GCX, which acts as a barrier between the blood and the vessel wall. This membrane-bound component of the endothelial GCX comprises PGs, GAGs, glycoproteins and glycolipids. GAGs are the major contributors to the structure and function of the endothelial GCX, including its mechanoregulatory roles. In particular, GAGs such as HS and HA constitute up to 90% of the endothelial cell GCX [100]. A necessary process in inflammation is the recruitment of leukocytes, guided by the chemokine gradients displayed by the endothelium. Leukocyte recruitment enhances the process of endothelial cell activation, which presents adhesion molecules to rolling leukocytes for adhesion and diapedesis. Indeed, HSPGs at the endothelial level are ligands for L-selectin expressed on leukocytes, transport chemokines in a controlled direction (basolateral to apical) across the endothelium, and present them at the luminal surface of the endothelium [275]. GAGs, such as HS and CS, mediate this chemokine immobilization, e.g., neutrophil trafficking is mediated by CXCL8 binding to GAGs. Several years ago, it was reported that GAGs are highly involved in endothelial downstream signaling in neutrophil recruitment during inflammation [276].

Leukocytes are also equipped with a thin, negatively charged GCX with a thickness of about 15 nm. As a result, the endothelial GCX may downregulate leukocyte adhesion by electrostatic repulsion on resting cells. When the HA or HS components of the endothelial GCX were removed by exogenous enzymatic action, leukocyte binding to endothelial cells was enhanced [277]. The surface GCX on activated endothelial cells is involved in adhesive interactions. When endothelial cells were stimulated with TNF-α, there was a significant increase in detachment force and work in the process of leukocyte adhesion. Treatment with heparinase I and III significantly reduced the force to levels similar to resting HUVECs treated with the same enzymes. This suggests that HS and HSPGs will likely be the major ligands for leukocyte adhesion molecules providing the vertical force under TNF-α stimulation. HS has been shown to bind strongly to L-selectin and weakly to P-selectin [278]. In addition, TNF-α may induce modifications in HS and HA critical for the initial selectin-dependent tethering step of leukocyte adhesion to the endothelial wall.

There is a constant bidirectional communication between immune cells and the ECM. The ECM receives signals from immune cells, but also provides mechanical cues to execute and regulate complex processes, such as cellular activation, proliferation, and cellular differentiation, to maintain homeostasis. In response to various insults such as pathogen infection, diabetes-related inflammation, and/or other damage, immune cells infiltrate the altered tissue and synthesize ECM molecules such as glycoproteins (GPs), PGs, and GAGs to promote tissue healing. If these interrelated events do not occur to the proper extent, a pathological state begins, be it chronic inflammation, autoimmune diseases, or cancer [279].

HA degradation generates fragments that influence receptor-mediated signaling in an inflammatory environment. These fragments activate cell recruitment and differentiation, activate resident cells, and in tumorigenesis favor tumor growth, survival, and metastasis [119], while under physiological conditions, HA fragments restore normal tissue function [268,274]. Notably, exogenous treatment of cells with hyaluronidase removes the pericellular coat, demonstrating the critical role of HA in matrix integrity [280]. The stiffness of cross-linked HA gels plays a crucial role in cell adhesion and spreading in bioengineering applications [281]. This suggests that local variations in Young’s modulus of the native pericellular matrix may affect its signaling potential, influencing whether it promotes cell adhesion and spreading, induces cell detachment and rounding, or contributes to the metastatic potential of cancer cells. It is suggested that HA and associated PGs may play an essential role in mechanochemical signaling at the cellular level and contribute to tissue material properties by providing swelling pressure. The ability of HA to form cables and alter swelling properties suggests that it may mediate tension, shear, and compression signals in tissues. Consequently, structured HA can create a dynamic network through which cells can communicate and respond to external forces [281]. In addition, HA can significantly alter the viscoelasticity of the ECM. For example, it has recently been shown that culturing human lymphatic endothelial cells (LECs) to form lymphatic cord-like structures (CLSs) on viscoelastic supramolecular HA hydrogels is particularly effective. These hydrogels promote CLS formation by increasing the expression of vital lymphatic markers such as lymphatic vascular endothelial HA receptor (LYVE-1), podoplanin, and prox1 compared to static elastic hydrogels. The viscoelastic hydrogels facilitate lymphatic CLS formation by upregulating NRP2, VEGFR2, and VEGFR3, enhancing VEGF-C stimulation [282].

LYVE-1, the primary receptor for HA expressed on the endothelium of lymphatic vessels, is a major receptor for leukocyte trafficking [283] and is an immunohistochemical marker of lymphatic vessels. Thus, LYVE-1 is a lymphatic docking receptor for dendritic cells. It regulates their entry into peripheral lymphatic vessels and their migration to lymph nodes where dendritic cells will participate in immune activation. LYVE-1 coordinates macrophage trafficking, and a recent study has shown that macrophages are involved in the angiogenesis of wound healing and tissue remodeling in injured dental pulp tissue [284]. This viscoelasticity attributed to the HA matrix can significantly regulate leukocyte trafficking and the inflammatory response [282]. PGs, expressed on the cell membrane surface, contained in the extracellular environment, or attached to intracellular granules, are key molecules for homeostasis. GAGs are covalently attached to the core protein of PGs, so PGs have diverse structures in their amino acid sequence, size, and shape.

The transmembrane HSPGs syndecans 1, 2, and 4 and membrane-bound glypican 1 are the major PGs found in the GCX of endothelial cells [285] and have an important mechano-modulatory role [286]. When Florian et al. used heparinase III to selectively degrade the HS component of endothelial cell GAGs in vitro, they found that the normal production of NO induced by steady or oscillatory shear over 3 h could be inhibited entirely [287]. It was also shown that GCX disruption with heparinase completely prevented the characteristic endothelial cell elongation and shear orientation after 24 h [288]. Thus, changes in PG/glycosaminoglycan components under inflammation provide a mechanical stimulus that regulates immune and endothelial cell functions.

In addition to mechanical cues modulated by PGs, PG expression is regulated by shear stress when exposed to endothelial cells [289]. SLRP are highly involved in immune events within the PG family. For example, lumican, keratocan, fibromodulin, biglycan, and decorin, the major SLRPs within the ECM, can be released from a remodeling ECM or synthesized by activated fibroblasts and immune cells. SLRPs can interact with various innate and adaptive immune cells, such as neutrophils and antigen-presenting cells (both macrophages and dendritic cells), and they can also interact with adaptive immune cells, such as B and T lymphocytes [290]. Recent studies using single-cell RNA sequencing of lymph nodes have shown that certain populations of stromal reticular cells express SLRPs, which regulate the cytokine milieu and connective tissue integrity [290].

### 7.3. Contribution to Inflammatory Signaling Pathways

HA binds to its receptor CD44 in its monovalent or multivalent configuration. This receptor induces intracellular signaling mainly when it binds to HA in a multivalent configuration [291,292]. Recently, it has been shown in gastric cancer that the short oligomers of HA, 6.4 kDa, induce activation of the mitogen-activated protein kinase (MAPK)–ERK pathway and increase cancer aggressiveness and invasion [293]. In another disease, chronic prostatitis/chronic pelvic pain syndrome, the CD44–HA couple has also been shown to be involved. Recently, this inflammatory couple has been shown to induce T helper 1 cell differentiation and activation of the Akt/mTOR pathway. Therefore, the study identified CD44–HA and Akt/mTOR signaling as new targets in this inflammatory disease [294].

In various diseases, including cardiovascular, pulmonary, autoimmune, and cancer, PGs such as versican and HA increase their expression in the ECM together with tumor necrosis factor-stimulated gene 6 (TSG-6) and inter-alpha trypsin inhibitor (IαI). These complex interactions activate signaling pathways that induce the synthesis and secretion of pro-inflammatory cytokines such as TNFα, IL-6, and the nuclear factor kappa B (NF-κB) family of transcription factors, key triggers for multiple signaling pathways [295]. When studying the role of GAGs in non-alcoholic steatohepatitis and hepatocarcinoma (HCC), it was shown that GAGs produced in the absence of the tumor suppressor EXTL2 act as danger molecules (e.g., DAMPs) via TLR4. This process is controlled by the activation of the transcription factor NF-κB, which contributes to injury and inflammation [296]. In breast cancer, HA has been shown to trigger the Wnt/β-catenin pathway, which influences cancer stem cell stemness and activates mesenchymal transition and overall cancer cell aggressiveness [297]. Furthermore, recent studies have shown in metastatic breast cancer cells that HA activates the NF-κB pathway and increases the expression of IL-8, modulating both cancer stem cells and non-cancer stem cells [298]. At the same time, other groups have demonstrated activation of the AKT and ERK pathways in breast cancer [299].

The HA–CD44 couple influences cancer stem cells in head and neck squamous cell carcinoma. This interaction has been shown to activate the Stat-3/Nanog and JNK/c-Jun pathways, which affect stem cell transcription factors such as Nanog, Oct4, and Sox2, thereby maintaining stem cell properties [300].

In atherosclerotic processes, the vessel wall retains low-density lipoproteins to modify PGs. Bacterial infections produce LPS, which increases inflammation by inducing the Smad2 signaling pathway. In an in vitro model, LPS has been shown to stimulate GAG-synthesizing enzymes, and TLR4 mediates this effect. LPS induces phosphorylation of Smad2 via TAK-1 and MAPK pathways, which are associated with genes encoding GAG chain initiation and elongation [301].

### 7.4. Role in Tissue Repair, Fibrosis, and Resolution of Inflammation

The role of the ECM and mechanotransduction as essential signaling factors in fibrosis is increasingly recognized. In addition to the ECM’s function as a cellular environment, its stiffness can compress or stretch cells and induce chemical changes within them based on collagen levels, cross-linking, hydration, and other ECM components. Fibrosis is characterized by densely cross-linked, disorganized, and widely dispersed ECM accumulation. Mechanotransduction, a dynamic signaling process in which mechanical forces trigger cellular responses, is critical to how fibrosis, including in the uterus, affects cell growth [302]. Recognizing the essential role of ECM stiffness in fibrosis may open new avenues for treating this common condition.

Dermal fibrosis, characterized by the excessive accumulation of extracellular matrix in the dermis, affects millions worldwide, leading to reduced mobility and disfigurement. Fibroblast dysfunction is central to the development of dermal fibrosis, which several factors regulate. Recent studies suggest fibroblasts can promote matrix deposition and stiffening, further exacerbating their functional dysregulation. In addition, cross-linking enzymes secreted by fibroblasts enhance resistance to ECM degradation and increase tensile strength [303], creating a stiffened environment that promotes the irreversible progression of fibrosis. Indeed, this creates a positive feedback loop that ultimately leads to uncontrolled pathological fibrosis [304]. PGs, such as syndecans and CD44, are other mechanosensitive transmembrane molecules that act alongside focal adhesion signaling. In lung fibrosis, for example, it was initially discovered that matrix stiffness induces the nuclear translocation of YAP by upregulating CD44. This process requires RhoA activity and polymerization of the F-actin cytoskeleton, leading to fibroblast activation. Furthermore, colocalization of CD44 and β1 integrins in the cell membrane was observed, suggesting a synergistic role in promoting fibroblast activation [305].

Chronic liver disease or recurrent hepatocyte injury can lead to hepatic fibrosis, characterized by excessive accumulation of ECM proteins such as collagens, glycoproteins, and PGs in the liver. This alters the composition of the ECM, increasing matrix stiffness and disrupting mechanical homeostasis. This imbalance activates hepatic stellate cells (HSCs) and transforms them into myofibroblasts, proliferating and secreting even higher levels of ECM proteins. These proteins accumulate in the Disse space, leading to failed matrix regeneration, further altering ECM components and increasing stiffness, creating a vicious cycle [306]. PGs have been shown to enhance the remodeling capacity of liver tissue, and their expression is significantly increased in cirrhotic liver tumors, particularly perlecan and decorin. Knockout of perlecan and decorin also reduces ECM stiffness [306,307]. Agrin is a membrane HSPG that is proteolytically degraded and deposited into the ECM [308]. In addition, during the pathogenesis of liver fibrosis and liver cancer, the excessive accumulation of agrin, together with collagens, laminins, and elastin, contributes to the formation of a stiffer ECM [309]. Notably, matrix stiffness regulates the expression of MMP-9 and TIMP-1 in hepatic stellate cells to facilitate fibrosis [310]. HA, which is essential in determining the mechanical properties of the matrix, is actively synthesized during liver fibrosis. Its accumulation begins in the early stages and increases significantly in advanced fibrosis [311]. Indeed, HA is widely regarded as a biomarker that is elevated in the blood of patients with liver fibrosis and cirrhosis [311].

The endothelial GCX is also involved in fibrotic processes. The hydrophilic nature of this structure contributes to the lubrication of the vascular surface and influences mechanotransduction, vascular permeability, and leukocyte adhesion, thereby regulating various processes of fibrosis resolution. Heparanase is a key enzyme in GCX degradation, specifically cleaving HS chains within endothelial GCX PGs. Dysregulation of heparanase function has been implicated in conditions such as organ fibrosis, sepsis, and viral infections [312].

As discussed, the mechanical environment can significantly influence cellular behavior and provide insight into drug target identification. Given the increasing prevalence of chronic diseases associated with alterations in extracellular matrix composition and tissue biomechanics, research to identify drug targets and develop therapeutics that target these tissue changes will undoubtedly be a significant focus. This line of research will be crucial for modifying disease progression in fibrotic conditions and developing regenerative therapies, as tissue regeneration is closely linked to tissue biomechanics.

## 8. Therapeutic Targeting of GAG/PG-Mediated Mechanosensing

Over the last few decades, oncology has witnessed a rapid accumulation of knowledge about tumorigenesis as an intricate multifactorial process. This knowledge has highlighted tumor cells’ immune evasion and complex interaction with the tumor microenvironment. The accumulation of knowledge has been crowned in the last decade with a new chapter in oncological therapies, immunotherapies involving monoclonal antibodies against checkpoint inhibitors, adoptive T-cell transfer, cytokines, vaccines, oncolytic viruses, specific onco-vaccines, etc. Although the oncological armamentarium has expanded tremendously, there are still cancers and/or patients that are refractory to these newly approved therapies; thus, novel therapeutic targets from the tumor microenvironment could improve the clinical outcome of these patients [313,314]. The tumor microenvironment, with its extracellular matrix, is essential for balancing anti-tumor and pro-tumor immune responses. PGs on cell surfaces can modulate the expression and functionality of various immune molecules, e.g., cytokines, chemokines, growth factors, and adhesion molecules. Acting as signaling co-receptors, PGs can influence both the biological activities of cancer cells and the microenvironment, thereby modulating tumor progression/regression. Because of these properties, cell surface PGs may be critical pharmacological targets in cancer [315].

### 8.1. Current Approaches and Challenges in Therapeutic Interventions

As discussed above, mechanical cues, including extracellular matrix stiffness, tissue elasticity and viscosity, fluid shear stress, tensile force, and hydrostatic pressure, can trigger various biological processes that support development and tissue homeostasis and are involved in disease pathogenesis. Therefore, activation of mechanical stimuli can have beneficial effects, but on the other hand, exaggerated mechanical stimulation can lead to pathological problems, including inflammation, fibrosis, and tumorigenesis. While the links between mechanical stimuli and tissue homeostasis or disease have been identified, the regulatory mechanisms between these stimuli are not fully understood, and effective therapies targeting mechanical stimuli-related signaling are currently lacking.

### 8.2. Potential Impact on Cancer, Inflammation, and Other Diseases

Tissue mechanical properties can alter cancer cell functions (such as growth, migration, invasion, metastasis, and dedifferentiation) and differ between tumors and normal tissues. Mechanotransduction signaling cascades are therefore important therapeutic targets for cancer treatment.

Many studies have demonstrated the role of the ECM in tumorigenesis. The ECM provides a scaffold for cancer and stromal cells and a biological pool for cytokines and growth factors. As discussed, ECM stiffness modulates cancer cell functions, including growth, differentiation, adhesion, migration, invasion, metabolic reprogramming, and EMT [316,317]. For example, ECM stiffness modulates lipid bilayer motions and consequent ion channel activation, leading to biochemical signaling and facilitating cancer cell migration [318].

Recently, the role of mechanotransduction cues, including high ECM stiffness, interstitial fluid pressure, and increased mechanical forces, has been implicated in resistance to cancer therapy [319,320]. Resistance to standard chemotherapeutic agents, including mitotic inhibitors, platinum alkylating agents, antimetabolites, and topoisomerase inhibitors, has been correlated with mechanical signaling [321]. Mechanical signals can influence chemotherapy response by activating chemoresistance states such as EMT and cancer stemness. EMT-affected cells exhibit increased expression of drug efflux pump genes, resistance to cancer cell adhesion to ECM components such as collagen and fibronectin, or growth in a stiff matrix, which promotes chemotherapy resistance [322]. This ECM-mediated chemoresistance, known as cell adhesion-mediated drug resistance (CAM-DR), is driven by the activation of integrin signaling. Triggering this pathway results in overexpression of prosurvival and anti-apoptotic proteins, cell cycle arrest, modulation of drug efflux, and phenotypic changes in cancer cells, such as EMT or cancer stemness [323,324]. For example, it has been shown that gemcitabine resistance can be induced by the activation of checkpoint kinase 1 (CHK1) and regulation of the cell cycle via the MMP/ERK1/2 signaling pathway in pancreatic cancer cells grown in a 3D collagen matrix. In a breast cancer model, increased stiffness of fibronectin-coated substrates was also shown to facilitate DNA repair of double-stranded DNA breaks, impairing the efficacy of several drugs, including etoposide and cisplatin. This mechanism was regulated by MAP4K4/6/7 kinase and subsequent ubiquitin phosphorylation, which recruits H2AX to DNA-damaged sites to activate DNA repair mechanisms [325]. In addition, matrix stiffness can regulate ATP-binding cassette (ABC) efflux transporter activities in an α-integrin-dependent manner and reduce chemotherapy efficiency, apoptosis, anchorage-independent growth, and stem cell properties [326].

Indeed, specific integrin inhibitors in combination with chemotherapeutic agents have been evaluated in phase I and II clinical trials, with promising results in the cases of cilengitide, vitaxin, and dasatinib [327,328,329]. However, the benefit of double therapy on patient survival has been limited, highlighting the need for new therapeutic modalities. PGs have been identified as essential mediators of ECM stiffness and integrin activity, and PG-targeted therapies may be promising for cancer sensitization.

Increased arterial stiffness, a natural consequence of aging, has recently been identified as a crucial mechanical stimulus in atherosclerosis, in addition to shear stress and stretch from blood flow [330]. Mechanosensing is also strongly correlated with other diseases, including lung and skin fibrosis, where TRPV4 promotes lung and skin fibrosis by modulating matrix protein expression and myofibroblast differentiation [331,332].

TRPV1 affects atherosclerosis by regulating lipid metabolism, inflammation, foam cell formation, and smooth muscle cell proliferation. It also affects diabetes and blood pressure [333,334]. Although capsaicin, a TRPV1 agonist, reduces lipid retention, its therapeutic use is limited by high toxicity [335]. TRPV4 has a complex role in atherosclerosis, involving foam cell formation, endothelial dysfunction, and inflammation [336]. Blocking TRPV4 has shown promise in treating several diseases, including cardiovascular disease. For example, the TRPV4 blocker GSK2193874 protects against heart failure-related pulmonary edema in rodent models [337]. A similar antagonist, GSK2798745, was well tolerated in human heart failure patients and is awaiting further studies [338]. Despite these findings, effective TRPV4 inhibitors for atherosclerosis must be further explored [339].

## 9. Future Perspectives

### 9.1. Emerging Research Directions and Unanswered Questions

The multifunctional role of GAGs/PGs in homeostasis, whether related to specific diseases or normal healing processes, presents a potential target for therapeutic approaches. GAGs/PGs interact with various cells through chemokine receptors, modulating cell functions and the immune response during inflammation. They also influence the mechanical properties of the ECM and act as sensing structures that can affect the tumor microenvironment in tumorigenesis. These multifunctional molecules are significant mechanotransduction players with exquisite properties in translating mechanical cues to biochemical signaling and vice versa. However, the therapeutic use of GAGs/PGs faces challenges, as their mechanisms of action are not fully elucidated.

### 9.2. Technological Advancements Enabling Further Understanding of GAG/PG-Mediated Mechanosensing

Although the shapes of organisms are encoded in their genomes, the developmental processes that lead to the final shape of vertebrates involve constant feedback between dynamic mechanical forces and cell growth and motility. The discipline of mechanobiology, the process of mechanotransduction that converts mechanical stimuli into biochemical signals, has emerged as a field dedicated to the study of the effects of mechanical forces and geometry on cell growth and motility—for example, during the development of cell–matrix adhesion. Mechanotransduction plays a central role in various physiological processes, such as cell differentiation, proliferation, and migration. The complexity of mechanotransduction is studied at multiple levels, often generating large amounts of data. Mechanotransduction is essential for triggering signaling cascades following cell and environmental interactions. The continuous generation of force by cells and the ability of biomolecules to change shape in response to piconewton forces provide the molecular basis for mechanotransduction. This process influences the guidance cues cells receive and the information flow they generate, including the temporal and spatial properties of intracellular signaling cascades.

Mechanotransduction is essential for initiating signaling cascades following cell interactions with their environment. Continuous force generation by cells and the ability of biomolecules to change shape in response to piconewton forces provide the molecular basis for mechanotransduction, influencing the guidance cues cells receive and the temporal and spatial properties of intracellular signaling. Mechanosensing, studied at multiple levels, generates vast amounts of experimentally observable data. Integrating artificial intelligence (AI) into the study of mechanotransduction is a significant step forward, as it can process and analyze large amounts of complex data, provide real-time insights, and create predictive models. This promising approach allows us to define measurable parameters that describe cell structure and dynamics, leading to a more comprehensive understanding of mechanotransduction. Harnessing advances in high-throughput data collection, computer simulation, and AI is essential for creating practical and predictive models of cell signaling networks [340,341].

AI excels in data analysis and pattern recognition of large datasets from high-throughput screening methods used in mechanotransduction studies. AI can uncover patterns and correlations that may not be immediately evident through traditional analysis methods [342,343].

Machine learning models can predict gene and protein expression changes in response to mechanical stimuli, aiding in understanding mechanotransduction pathways and the localization of mechanosensitive factors.

In a recent study, researchers exploited microscale heterogeneity in engineered fiber microenvironments to generate a large dataset of cell morphologies and measure mechanobiological responses (YAP/TAZ nuclear localization) at the single-cell level across different cell types [344]. The extensive dataset of engineered fiber microenvironments was analyzed using machine learning to predict the mechanobiological state of single cells from different lineages. The study found that specific cells (e.g., invasive cancer cells) or biochemical changes (e.g., changes in contractility) could affect the predictability of these models [344].

To address this, models were developed that incorporated biochemical cues for single-cell prediction and identified cells that deviated from established patterns. They linked cell morphology and signaling, integrated biochemical cues into predictive models, and identified abnormal cell behavior at the single-cell level [345]. AI can develop predictive models to simulate the mechanical environment of cells and predict how changes in mechanical forces will affect cell behavior. This application of AI demonstrates its potential for modeling diseases related to abnormalities in mechanotransduction, such as cancer metastasis and fibrosis, by analyzing the mechanical properties of cells and tissues.

Alterations in nuclear morphology have long been a hallmark of cancer and a critical diagnostic tool for pathologists to assess the malignant potential of cancer cells. Mechanical forces exerted on surface-adhesion receptors like integrins and cadherins are transmitted along cytoskeletal filaments and focus upon remote locations in the cytoplasm and nucleus. In this context, extracellular forces can trigger mechanochemical reactions in the nucleus and modify gene activities [346]. Traditionally, pathologists have focused on visible defects in nuclear and chromatin structures, but the heterogeneous nature of tissue environments and subjective interpretation limits this method.

Recent advances in imaging and machine learning have introduced new methods to address these limitations. Parametric machine learning techniques use quantitative nuclear morphometric data such as size, shape, nucleus-to-cytoplasm ratio, and chromatin texture to classify histopathology images. Non-parametric methods such as deep learning have been used to diagnose cancers, including prostate cancer, with high accuracy [347]. These techniques learn features directly from images, which can include staining for DNA, transcription factors, DNA repair proteins, nuclear bodies, nuclear lamina, or specific genes and chromosomes.

AI algorithms can analyze live cell imaging data in real time, providing immediate feedback on cellular responses to mechanical forces and enabling dynamic adjustments to experimental conditions. Technological advances have transformed tumor models from simple two-dimensional cell cultures to sophisticated three-dimensional printed models with greater complexity and variable performance. These new models more closely mimic the architecture and heterogeneity of the tumor microenvironment. AI integration enables high-throughput systems for real-time monitoring of tumor growth and biophysical properties, enhancing the potential for personalized medicine through AI-assisted tumor modeling [348]. Recently, a novel method for assessing ovarian cancer tissue heterogeneity using image processing techniques and AI proposed a classification system based on radionics [349]. Another implementation of AI is in drug development. Notably, a machine learning approach was utilized to estimate the changes in cell mechanical stress when exposed to drugs [350].

**Figure 8:**
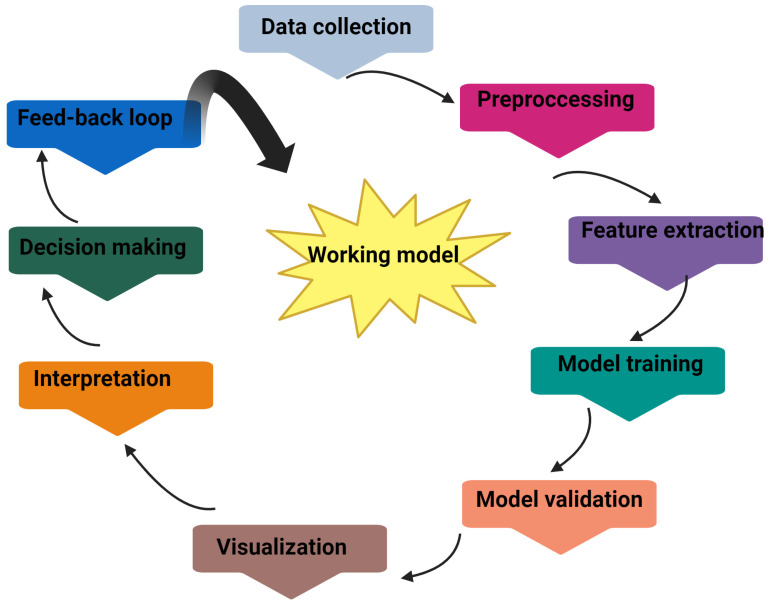
A standard pipeline implementing machine learning/deep learning to interpret and assess mechanotransduction cues. Created in BioRender. Nikitovic, D. (2024). BioRender.com/m58b525 (accessed on 12 September 2024).

Ensuring high-quality, standardized data is critical for AI applications in mechanotransduction, as variability in experimental conditions and data acquisition methods can affect the performance of AI models. In this context, the organization and exchange of data should follow the four guiding principles of findability, accessibility, interoperability and reusability (FAIR) to feed and train machine learning-based applications. In summary, AI is critical to understanding mechanotransduction in biology due to its capabilities in data analysis, real-time monitoring, predictive modeling, integration with advanced technologies, improvement of drug screening, standardization of data, and promotion of interdisciplinary collaboration. Together, these capabilities enhance our ability to study and manipulate mechanotransduction pathways, leading to new insights and potential therapeutic strategies. The implementation of AI for data assessment is presented in Figure 8.

## 10. Conclusions

Mechanotransduction regulation is another emerging facet of the multifunctional GAG/PG roles. GAGs/PGs modulate the mechanical properties of ECM, but are also sensing structures that can participate in signal transmission and regulation of cell behavior. Even though major advancements have been made in the last few years in understanding ECM–cell mechanotransduction, especially regarding the role of integrin and mechanosensitive ion channels, critical gaps in our comprehension remain. Thus, the roles of downstream-involved effectors, e.g., subcellular cytoskeletal structures, signaling pathways, and transcription factors, are not fully understood.

Emerging tools such as super-resolution imaging revealing the structures and dynamics of integrin-based adhesions, the actin cytoskeleton, actomyosin machinery, and actin adaptor proteins like talin and vinculin, which are correlated with GAG/PG mechanosensing, can advance this direction of research. Furthermore, genome-wide assays such as RNA-seq or single-cell RNA-seq can identify unique, GAG/PG-associated mechanotransduction regulators. The central role of GAGs/PGs in the complex process of inflammation, whether associated with a specific disease or within the normal healing process, represents a potential target for specific therapeutic approaches. Likewise, ECM characteristics modified by GAGs/PGs, such as stiffness, are starting to be clinically targeted in cancer. Along these lines, β1 integrin-mediated mechanotransduction is currently being evaluated in a clinical trial (NCT02683824). Therefore, issues to be clarified in therapeutic assessments, such as modulating dense or fibrotic tissue or efficiently passing the blood–brain barrier in neurodegenerative disorders, are immediately correlated with advancing the knowledge on GAG/PG mechanical cues.

## Figures and Tables

**Table 1:** Molecules regulating ECM mechanical properties.

Stiffness/Elasticity	Viscoelasticity	Strength & Toughness	Anisotropy	Adhesive Properties	Remodeling & Plasticity
Collagen	Collagen	Collagen	Collagen	Collagen	Cell-matrix adhesion receptors
Elastin	Elastin	Elastin	Elastin	PGs	Cytokines & growth factors
Glycoproteins	Glycoproteins	Glycoproteins	Glycoproteins	Glycoproteins tenascin	TGF-b
Crosslinking molecules	Crosslinking molecules	Crosslinking molecules	Crosslinking molecules	Fibronectin	Fibroblasts & myofibroblasts
PGs	PGs	PGs	PGs	Laminin	Crosslinking enzymes
Water	Water	Water	Water	Integrins	Matrix metalloprorteinases
Matrix metalloproteinases	GAGs			GAGs	Tissue inhibitors of MMPs

## Abbreviations

ABS	actin-binding site
AFM	atomic force microscopy
AGE	advanced glycosylation end product
AI	artificial intelligence
Arf6	ADP-ribosylation factor 6
ARP	actin-related protein
BBB	blood–brain barrier
CD44	cluster of differentiation 44
CDH	cadherin
CLS	cord-like structures
CREB	cAMP response element-binding protein
CS	chondroitin sulfate
CSPGs	chondroitin sulfate proteoglycans
CXCL	chemokine (C-X-C motif) ligand
Da	Daltons
DAMP	damage-associated molecular pattern
DCCM	directional collective cell migration
DDR	discoidin domain receptor
DS	dermatan sulfate
ECM	extracellular matrix
eNOS	endothelial nitric oxide synthase
ERK1/2	extracellular signal-regulated kinase 1/2
FAK	focal adhesion kinase
FAT domain	focal adhesion targeting domain
FERM	F for protein 4.1, E for ezrin, R for radixin, and M for moesin
FSS	fluid shear stress
GAGs	glycosaminoglycans
Gal	galactose
GBM	glioblastoma multiforme
GCX	cellular glycocalyx
GPCR	G protein-coupled receptors
H1Rs	histamine H1 receptors
HA	hyaluronan
HCC	hepatocarcinoma
Hep	heparin
HS	heparan sulfate
HSPG	heparan sulfate proteoglycan
HSCs	hepatic stellate cells
IGF-IR	insulin growth factor I receptor
IL	interleukin
KLF	Krüppel-like factor
KS	keratan sulfate
LOX	lysyl oxidase
LOXLs	lysyl oxidase-like proteins
LYVE-1	lymphatic vessel endothelial hyaluronan receptor 1
MAPK	mitogen-activated protein kinase
MMPs	matrix metalloproteinases
MT1-MMP	membrane type I matrix metalloproteinase
NF-κB	nuclear factor κB
NRP	N-rich protein
PAMP	pathogen-associated molecular pattern
PECAM	platelet and endothelial cell adhesion molecule
PGs	proteoglycans
PI3K	phosphatidylinositol 3-kinase
PIP2	phosphatidylinositol 4,5-bisphosphate
PRELP	proline/arginine-rich end and leucine-rich protein
RHAMM	hyaluronan-mediated motility receptor
ROCK	Rho-associated protein kinase
SLRP	small leucine-rich proteoglycan
TAZ	transcriptional co-activator with PDZ-binding motif
TEAD	transcriptional enhanced associate domain
TGFβ	transforming growth factor beta
THD	talin head domain
TLRs	Toll-like receptors
TNBC	triple-negative breast cancer
TNF	tumor necrosis factor
TRP	transient receptor potential family
TRPV4	transient receptor potential cation channel, subfamily V, member 4
TSG-6	tumor necrosis factor-stimulated gene 6
VBSs	vinculin-binding sites
VEGF	vascular endothelial growth factor
WISPs	WNT1-inducible signaling pathway proteins
YAP	yes-associated protein
